# Chromatin modifiers in human disease: from functional roles to regulatory mechanisms

**DOI:** 10.1186/s43556-024-00175-1

**Published:** 2024-04-08

**Authors:** Yali Nie, Chao Song, Hong Huang, Shuqing Mao, Kai Ding, Huifang Tang

**Affiliations:** 1 Hunan Provincial Key Laboratory of Multi-omics and Artificial Intelligence of Cardiovascular Diseases, University of South China, Hengyang, Hunan 421001 China; 2 The First Affiliated Hospital, Department of Cardiology, Hengyang Medical School, University of South China, Hengyang, Hunan 421001 China; 3 The First Affiliated Hospital, Institute of Cardiovascular Disease, Hengyang Medical School, University of South China, Hengyang, Hunan 421001 China; 4 Clinical Research Center for Myocardial Injury in Hunan Province, Hengyang, Hunan 421001 China; 5 The First Affiliated Hospital, Cardiovascular Lab of Big Data and Imaging Artificial Intelligence, Hengyang Medical School, University of South China, Hengyang, Hunan 421001 China

**Keywords:** Chromatin modifiers, Human disease, Transcriptional regulation, Therapy

## Abstract

The field of transcriptional regulation has revealed the vital role of chromatin modifiers in human diseases from the beginning of functional exploration to the process of participating in many types of disease regulatory mechanisms. Chromatin modifiers are a class of enzymes that can catalyze the chemical conversion of pyrimidine residues or amino acid residues, including histone modifiers, DNA methyltransferases, and chromatin remodeling complexes. Chromatin modifiers assist in the formation of transcriptional regulatory circuits between transcription factors, enhancers, and promoters by regulating chromatin accessibility and the ability of transcription factors to acquire DNA. This is achieved by recruiting associated proteins and RNA polymerases. They modify the physical contact between cis-regulatory factor elements, transcription factors, and chromatin DNA to influence transcriptional regulatory processes. Then, abnormal chromatin perturbations can impair the homeostasis of organs, tissues, and cells, leading to diseases. The review offers a comprehensive elucidation on the function and regulatory mechanism of chromatin modifiers, thereby highlighting their indispensability in the development of diseases. Furthermore, this underscores the potential of chromatin modifiers as biomarkers, which may enable early disease diagnosis. With the aid of this paper, a deeper understanding of the role of chromatin modifiers in the pathogenesis of diseases can be gained, which could help in devising effective diagnostic and therapeutic interventions.

## Introduction

Dynamic changes in chromatin state result in unique expression patterns of genes during development. Chromatin modifiers in open regions of chromatin assist in forming transcriptional regulatory circuits among transcription factors, enhancers, and promoters. This regulation has a significant impact on chromatin accessibility and the ability of transcription factors to acquire DNA, which can affect human health and disease [1]. There are various types of human diseases that undergo complex pathological changes and processes. Recently, there has been a significant amount of evidence that abnormal gene transcriptional regulation processes are responsible for the occurrence of diseases. However, the pathogenesis of human diseases still lacks adequate understanding in our current knowledge. It is increasingly evident that abnormal perturbations of chromatin modifiers are strongly linked to disease, indicating the need for further exploration of potential mechanisms by which chromatin modifiers are involved in human diseases [2, 3]. Such exploration can beneficial for early prevention, diagnosis, and treatment of diseases, thereby reducing the risk of disease incidence and mortality.

**Fig. 1:**
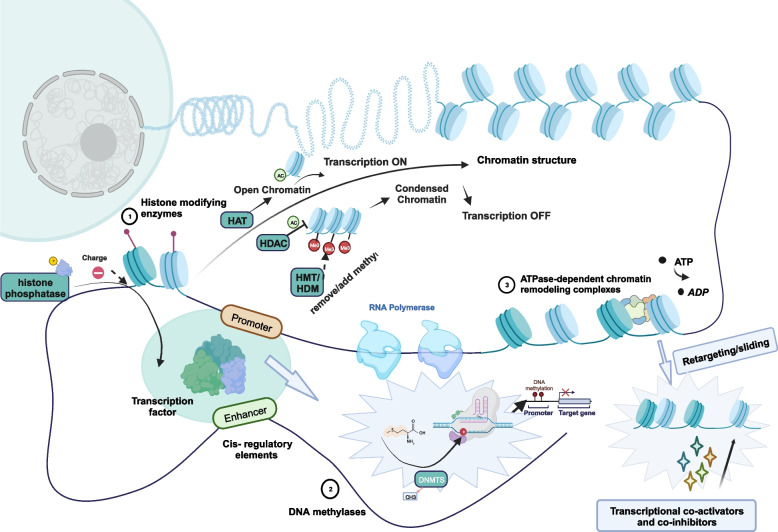
An overview of the regulation of genes by chromatin modifiers. Chromatin modifiers are involved in cardiac transcriptional regulatory networks, as follows: Histone Acetyltransferase (HAT) loosens the DNA to the nucleosome, allowing promoters to enter, promoting transcriptional activation, and thus initiating gene expression. HDAC regulates chromatin concentration and denies promoter entry and thus transcriptional regulation. Histone phosphorylase adds a negative charge to histones, enhances the interaction of some regions of chromatin with transcription factors, and adjusts the fine structure of chromatin, thereby affecting transcriptional regulation. HMT assists histone methylation and plays a role in maintaining chromatin status and gene suppression. Histone demethylase (HDM) exerts demethylation function and activates transcriptional regulation and expression of related genes. DNMT assists in DNA methylation modification by transferring the methyl group of S-adenosylmethionine to the base of cytosine by covalent modification. ATPase-dependent chromatin remodeling complexes use the energy released by ATP to subject the local or global chromatin structure to non-covalent regulation. This allows the sliding or repositioning of nucleosomes in a dynamically changing state to recruit transcriptional coactivators and co-repressors for DNA promoters, thereby regulating transcriptional processes

Chromatin modifiers are a category of enzymes that play a significant role in DNA and histones. They help catalyze the chemical conversion of pyrimidine and amino acid residues, such as cytosine in DNA and tyrosine, lysine, serine, and arginine in histones. A growing number of studies have explored the characterization of chromatin modifiers and their role in disease development. However, it is crucial to note that there is a need for more comprehensive and precise elucidation of these modifiers. Moreover, they have been shown to be critical elements of transcriptional regulatory circuits, which fall under trans-regulatory elements. The classification of chromatin modifiers includes DNA methyltransferases (DNMTs), histone modifiers such as histone methyltransferases (HMTs) and histone deacetylases (HDACs), and chromatin-remodeling complexes [4]. In addition, chromatin modifiers do not affect transcriptional regulation processes by changing the position and size of the DNA sequence. Instead, they function by recruiting associated proteins and RNA polymerases to alter the degree of physical contact between cis-regulatory factor elements, transcription factors, and chromatin DNA. Chromatin modifiers work with transcription factors, transcriptional cofactors, CTCF, and DNA-related regulatory elements to regulate important gene expression throughout each growth cycle and differentiation. Studies have shown that chromatin modifiers have the characteristics of “readers”, “writers”, and “erasers”. They can bind to enhancers, co-regulating super-enhancers, and are linked to chromatin remodeling, which transforms chromosomes into transcriptionally active or inactive chromatin, causing changes in the chromatin’s composition and structure [5, 6] (Fig. 1).

Chromatin modifiers are incorrectly regulated in the presence of specific triggers, which affects gene transcription and promotes the occurrence of diseases [7, 8]. For example, the deletion of KMT5B (HMT) in muscle stem cells promotes the transcription of DNA in the S phase, induces the accumulation of abnormal DNA hybrids and transcription-replication conflict in oncogenes, thereby promoting the continuous activation and proliferation of quiescent muscle satellite cells and causing the formation of rhabdomyosarcoma [9]. Similarly, chromatin remodeling is indispensable for regulating cardiovascular development and disease pathogenesis. For instance, the interaction of enhancers with promoters of genes related to developmental regulation can be facilitated by histone acetyltransferase P300, which impacts mouse cardiomyocytes’ reprogramming and proliferation [10]. Chromatin remodeling can also regulate the level of lactation after myocardial infarction, participate in myocardial remodeling, and regulate the level of pyruvate metabolism of myocardial cells to affect myocardial hypertrophy [11]. This series of evidence has shed light on the critical role of chromatin modifiers in the development and progression of human disease and has raised new understanding of chromatin modifiers. Furthermore, drugs targeting chromatin modifiers have been tested in animal models of various diseases, and some have been applied in clinical treatment. Also, the specific roles of these drugs need further clarification.

In this review, we describe the link between chromatin modifiers and human diseases using cardiovascular disease as the central example and aim to elucidate their disrupting or protective effects on essential regulatory genes in these diseases. We also reviewed recent insights into the regulatory mechanisms of transcriptional regulatory circuits by chromatin modifiers. Finally, we discuss the main drug applications of targeted chromatin modifiers, hoping that the emergence of new therapeutic strategies will provide new prospects for the prevention, diagnosis, and treatment of human diseases.

## Role of chromatin modifiers in human diseases

Dysregulation of chromatin modifiers has been documented in various disease models, such as cardiovascular diseases, cancers, neurological diseases, and immune diseases. It is worth noting that maintaining human health and stability requires cooperation between chromatin modifiers and other transcriptional regulatory elements. Chromatin modifiers exhibit characteristic changes when exposed to external stimuli: they affect chromatin accessibility to increase or decrease the degree of physical contact between transcriptional regulatory circuits and chromatin DNA. Such alterations may increase the risk of related diseases, affect disease prognosis, and contribute to organ function. Therefore, it is essential to identify the regulatory mechanisms and targets of chromatin modifiers involved in major human diseases.

### Cardiovascular diseases

Several recent studies have highlighted the connection between abnormal regulation of chromatin modifiers and the development of cardiovascular disease. Cardiovascular disease is a major public health issues that poses a risk to human life and well-being. At present, the prevention model aims to effectively reduce or delay the incidence of cardiovascular events by intervening in daily life and controlling risk factors. Studies have shown that chromatin modifiers may promote the occurrence and progression of cardiovascular diseases by influencing pathophysiological mechanisms such as glycolipid metabolism, oxidative stress, inflammatory response, and programmed cell death. Therefore, timely intervention in the aberrant regulation of chromatin modifiers may delay the onset of cardiovascular diseases.

#### Atherosclerosis

The characteristics of atherosclerosis include macrophage movement, smooth muscle cell proliferation, connective tissue matrix formation, and lipid accumulation. It is noteworthy that chromatin modifiers can be present in lipid infiltration, endothelial injury reactions, or other atherosclerotic pathogenic pathways.

Chromatin modifiers aim to regulate atherosclerotic plaque hyperplasia and disorders of lipid metabolism. According to recent studies, the FOXP3 gene of regulatory T cells is modified by DNMT3b, with an increased level of methylation, thereby affecting the content of oxidized low-density lipoproteins in the blood and vascular inflammation, reducing the proportion of collagen and smooth muscle cells in blood vessels, and ultimately promoting atherosclerosis [12, 13]. In addition, it is proved that a specific bone marrow DOT1L knockout can affect the biosynthesis of fatty acids and cholesterol in macrophages, of which Srebf1 and Srebf2 (sterol-regulated binding factors) were the most inhibited; the DOT1L inhibitor SGC0946 (clinical EPZ-5676) has also been shown to have the same effect in human and mouse macrophages [14]. The study also proved that DOT1L deficiency could cause inflammatory storms and aggravate the formation of inflammatory plaques. Past’s study reported that the Baf60a (Baf subunit of the ATP chromatin remodeling complex SWI/SNF)-PPARα-PGC1α transcriptional complex regulates fatty acid oxidation mechanisms in hepatocytes [15]. In mice with Baf60a knockout fed with a high-fat diet, Baf60a affects the occurrence of atherosclerosis by feedforward regulating bile acid metabolic homeostasis and cholesterol absorption [16].

Chromatin modifiers target vascular smooth muscle cell function and vascular shear stress to expedite disease development. As evidenced by previous studies, HDAC3, 5, and 7 have been identified as being responsible for promoting oscillatory shear stress in atherosclerotic endothelial cells and inhibiting the shear flow pattern of protective pulsating shear stress [17, 18]. Angiotensin II (Ang II) promotes HDAC3 to support the TGFβ (transforming growth factor β)—P38 (mitogen-activated protein kinase) pathway to inhibit PPARγ expression and accelerate lipid invasion in vascular smooth muscle cells [19]. In healthy blood vessels, focal adhesion kinase (FAK) is present mainly in vascular smooth muscle cells and tissues, where it lowers the stability of DNMT3A, increases the vascular smooth muscle contraction phenotype, and maintains arterial plasticity. However, the dedifferentiation of vascular smooth muscle is accompanied by a decrease in FAK expression in cells and tissues and an increase in DNA methylation in diseased arteries [20].

Chromatin modifiers also target inflammation and oxidative stress in vascular endothelial injury. Recent research has shown that DOT1L changes the genomic accessibility of NF-kB, regulates the expression of the downstream inflammatory factors CCL5 and CXCL10 of the NF-kB pathway, and prevents the occurrence of atherosclerosis in mice [21]. The HMT SETDB2 is expressed abundantly in the proinflammatory macrophages of atherosclerotic plaques and is lost in hematopoietic cells, exacerbating vascular inflammation [22]. High methylation levels of the dyferlin gene’s promoter activate its transcription, enhancing monocyte activation in atherosclerosis [23]. Atherosclerotic tissues are stimulated to produce inflammatory factors like interleukin-1 by SNF5 (a part of the SWI/SNF complex) [24]. Homocysteine induces the expression of DNMT1 in extracellular superoxide dismutase, thereby inhibiting the expression of extracellular superoxide dismutase and increasing the level of arterial oxidative stress, ultimately promoting the development of atherosclerosis [25]. Another study showed that after overexpression of DNMT1, Rnase6, which was originally upregulated in peripheral blood samples and plaques, was downregulated, and the PI3K/AKT/mTOR signaling pathway, in which Rnase6 participates in activation, was inhibited; however, the area of atheroma was reduced [26]. It can hypothesized that chromatin modifiers can lead to different cell fates when assisted by diverse transcription factors.

#### Myocardial infarction

Myocardial infarction (MI) poses a high risk of mortality and disability, which is a threat to human safety and quality of life. Small extracellular vesicle (EVs) that promote neovascularization after myocardial infarction can lose their original cardioprotective effects in mice with diabetes and myocardial infarction due to decreased H3K9Ac levels and increased HDAC enzyme activity [27]. Moreover, high expression levels of DNMT3A and DNMT3B and reduced expression of TET genes that contribute to demethylation can detected in plasma-isolated and purified EVs of patients with acute coronary syndrome [28]. DYRK1A (bispecific tyrosine regulatory kinase 1A), which has the ability to regulate blood glucose, has been found to phosphorylate HAT KAT6A and HMT WDR82 in myocardial infarction, thereby inhibiting histone acetylation and methylation levels of cell cycle-related genes and hindering post-myocardial infarction cardiac repair [29–31]. Persistent myocardial hypoxia can exacerbate myocardial necrosis after MI. The expression of DNMT1 and DNMT3B in human primary fibroblasts increased in hypoxia, and the fibroblast phenotype changed to synthetic, which increased the gene expression levels of a-SMA and collagen to promote cardiac fibrosis [32]. UCP2 (mitochondrial uncoupling protein 2) can target Hat1 expression and inhibit histone deacetylase in hypoxia, which leads to a rise in cardiomyocyte energy metabolism and protection of cardiac functions [33].

Chromatin modifiers are directed toward signaling pathways like cardiomyocyte oxidative stress, myocardial energy metabolism, and programmed cell death, which contribute to the pathogenesis of myocardial infarction. The myocardial infarction zone lacks sufficient energy supply, which exacerbates the injury response of myocardial infarction. Studies have shown that treatment with sodium caprylate (medium-chain fatty acid) in rats with myocardial infarction can promote HAT KAT2A to increase the level of H3K9ac at the promoter of HO1 and NQO1 genes, improving the antioxidative stress and anti-apoptosis ability of cardiomyocytes [34]. Histidine decarboxylase (associated with immunity and ROS production) restricts the transcriptional expression of the HMT PRMT1 by recruiting SWI/SNF complexes, thereby avoiding myocardial damage caused by massive ROS production [35]. Moreover, the expression of HDM KDM6A markedly elevates in myocardial infarction tissues, and blocking KDM6A could prevent sodium-calcium exchange and cardiomyocyte apoptosis in myocardial infarction rats [36]. The upregulation of KMT2B can activate the expression of NOX2 (nicotinamide adenine dinucleotide phosphooxidase 2), a key gene of multiple signaling pathways, thereby enhancing cardiomyocyte apoptosis and expanding the infarct area [37]. KDM3A regulation of the pyroptosis pathway has been suggested to alleviate ischemia/reperfusion injury in cardiac microvessels [38].

The objective of chromatin modifiers is to target cardiac remodeling and disease prognosis after myocardial infarction. A recent targeted deep sequencing study showed that approximately 10% of patients diagnosed with ST-elevation myocardial infarction carry mutations driven by the TET2-CH and DNMT3A alleles. There is a correlation between the changes detected in the plasma samples of these patients and their prognosis, thus identifying new risk factors for cardiovascular disease [39]. The lncRNA ZFAS1 has been identified to have apparent differential expression in myocardial infarction, which binds to the promoter of Notch1 and recruits DNMT3B to the promoter of Notch1, triggering Notch1 methylation and inhibiting the expression of Notch1, hindering cardiac repair and cardioprotective function [40, 41]. Heart repair occurs almost simultaneously with heart damage. Myocardial fibrosis is exacerbated after myocardial infarction and manifests as the differentiation of cardiac fibroblasts into myofibroblasts. The down-regulation of DNMT1 expression and the expression of myofibroblast marker a-SMA were detected in the myocardial infarction region, but the degree of a-SMA methylation decreased. When DNMT1 is overexpressed, it causes an increase in a-SMA methylation level and inhibits the differentiation of cardiac fibroblasts into myofibroblasts, which improves cardiac fibrosis [42]. MI rats upregulate cardiomyocytes’ HMT Smyd1 expression after regular exercise to promote the optimization of pathological remodeling of cardiomyocytes, resulting in compensatory hypertrophy of cardiomyocytes and reducing the level of oxidative stress [43]. Therefore, it can be considered that regular cardiac rehabilitation exercises in clinical practice will be beneficial to the prognosis of patients with MI.

#### Hypertension

The occurrence and development of hypertension involve various factors, polygenes, and multi-link interactions. Chromatin modifiers geared toward regulating hypertension with the renin–angiotensin–aldosterone system. In hypertensive mice treated with Ang II, HDAC5 deletion significantly reduced systolic blood pressure and inhibited vascular smooth muscle cell contraction [44]. Moreover, exposing a fetus to timed glucocorticoids in utero increases the effects of HDAC and DNMT on oxidative stress, affecting fetal cardiovascular gene programming and increasing the risk of hypertension in the growing fetus [45, 46]. The participation of DNMT3A in the prenatal accumulation of glucocorticoids in pregnant women can increase the chance of hypertension in offspring. In the disease mechanism, DNMT3A expression decreases the inhibition of methylation of the promoter of angiotensin receptor 1a while increasing DNA demethylation and promoting the expression of angiotensin receptor 1a [12]. This genetic change is inherited by offspring, thereby increasing their genetic susceptibility. In addition, prenatal exposure of pregnant women to inflammatory stimuli can cause increased expression of DNMT1 and DNMT3B in the offspring, increased expression of inflammatory cytokines, and increased prevalence of hypertension in the descendant [47].

Chromatin modifiers can have an impact on survival and prognosis by affecting hypertension’s risk factors and target organ function. A long-term high-salt diet will increase the burden on blood vessels and is a risk factor for hypertensive diseases. Therefore, a cumulative regulation of chromatin modifiers must occur for the genes involved in such eating habits. Yueyuan et al. screened 152 participants in a chronic salt-loading diet intervention study to participate in a follow-up survey. A study found that the expression of the HMT Set7 and the HDM LSD1 in the serum of the salt-sensitive human group was upregulated, and the blood pressure increased compared to the baseline level of the normal group [48]. Set7 and LSD1 are speculated to be involved in the development of salt-sensitive hypertension. Of concern is that prolonged intermittent hypoxia inhibits HDAC activity [49]. Especially for patients with obstructive sleep apnea syndrome, it increases the likelihood that they will have high blood pressure. Previous studies have demonstrated that the CHD family proteins CHD1L and CHD9 promote phenotypic conversion and proliferation of vascular smooth muscle cells in hypertensive patients, vascular oxidative stress, and remodeling [50, 51]. G protein-coupled receptor 37L1 expressed at the brush border boundary of proximal tubular cells in the kidney inhibits DNMT1 expression through the PI3K/AKT/mTOR signaling pathway, resulting in NHE3 promoter methylation (sodium proton exchanger 3) disorder. This process regulates blood pressure by promoting NHE3 expression and increasing sodium transport in the renal lumen [52]. It can be concluded that DNMT in the kidney can regulate the DNA methylation of genes related to hypertension and inhibit the transcription initiation of related genes, thereby promoting or inhibiting the progression of hypertension.

#### Myocardial hypertrophy

The protective or damaging effects of chromatin modifiers on myocardial hypertrophy can be altered by changes in cellular metabolism and neuroendocrine hormone levels, as supported by strong evidence. High expression of HAT P300, HMT NSD2, and DNMT1 was detected in a model of myocardial hypertrophy induced by various factors, such as high glucose and phenylephrine levels [53–56]. This suggests an association with the development of myocardial hypertrophy. The expression of Brg1 in both TAC and isoprenaline-induced cardiac hypertrophy models was upregulated, demonstrating that hypertrophy signaling promotes the enrichment of Brg1 to regulate the transcriptional activator of the pro-hypertrophy gene, GATA4, and promote cardiac hypertrophy [57]. Previous studies demonstrated that docosahexaenoic acid and eicosapentaenoic acid directly inhibit the activity of P300, prevent the hypertrophic response of cardiomyocytes after P300-induced myocardial infarction, and improve cardiac function [58]. The hypoglycemic drug Metformin can also inhibit the progression of myocardial hypertrophy by inhibiting P300-regulated histone acetylation, but overexpression of P300 can reverse the protective effect of Metformin on the heart [59]. P300 can also form a complex with GATA4 to increase the transcriptional regulation of cardiac hypertrophy genes and promote the occurrence of myocardial hypertrophy [60]. But another HMT SUV39H1 is recruited to the GATA4 promoter and interacts with kindlin-2 (an integrin-interacting protein) to inhibit the expression of GATA4, protecting the heart from hypertrophy [61]. In addition, the HMT SET1 is activated by angiotensin II stimulation and recruited to the promoter of endothelin-1(a potent vasoconstrictor) to initiate the transcription of endothelin-1 and promote pathological myocardial hypertrophy [62]. PRMT5-regulated histone H4R3me2s promoted cardiac hypertrophy after impairment of isoprenaline-induced cardiac hypertrophy, but overexpression of PRMT5 reversed myocardial hypertrophy [63].

Chromatin modifiers have an impact on myocardial hypertrophy by affecting the level of epigenetic modifications. Numerous studies have confirmed that class II HDACs (HDAC4, 5, 7, 9, 10) inhibit the development of myocardial hypertrophy, while class I HDACs (HDAC1, 2, 3, 8) have a positive effect on myocardial hypertrophy. Polyunsaturated fatty acids have also been reported to protect the heart. For example, the lack of HDAC4 and HDAC5 leads to the weakening of cAMP-driven inhibition of cardiomyocyte hypertrophy [64]. HDAC5 also inhibits the transmission of oxidation–reduction signals to inhibit the transcription of ROS-stimulated NRF2-mediated promyotrophic signaling pathways [65]. After the physical binding of protein phosphatase 2A to HDAC2 to form a functional complex, HDAC2 is regulated by dephosphorylation, which inhibits the hypertrophy-promoting effect of HDAC2 [66]. Research suggests that the HMT EZH2 is involved in maintaining cardiac homeostasis. Upregulated hypertrophy-related and profibrotic genes can be detected in cardiomyocytes lacking EZH2 [67]. EZH2 also is a target for many noncoding RNAs involved in regulating myocardial hypertrophy [68–70]. DNMT3 is indispensable for developing cardiac hypertrophy. Although mice with DNMT3A deletion in cardiomyocytes showed smaller cardiomyocyte morphology, hypertrophy-related genes were activated again [71], indicating that the loss of DNMT3A did not lead to the complete disappearance of cardiac hypertrophy. Overexpression of NSD3 can inhibit atrial natriuretic factor transcription, exert cardioprotective effects, and inhibit the development of cardiac hypertrophy [72]. In addition, the deletion of the HDMs KDM3A or JMJD2A and the silencing of JMJD3 have been shown to attenuate cardiac hypertrophy responses [73–75]. It is worth mentioning that if cardiomyocyte-specific lack the insulator CTCF, it may promote the disappearance of chromatin loops with enhancer-promoter interactions, leading to the development of dilated cardiomyopathy [76]. This loss causes alterations in chromatin structure, which is dynamic in nature and induces aberrant regulation of genes associated with cardiac hypertrophy. This phenomenon is hypothesized to be related to histone methylation and DNA methylation.

#### Heart failure

Heart failure is not an isolated disease but an end stage of various heart diseases. Multiple demonstrations have shown that chromatin modifiers are powerful candidate therapeutic intervention targets and diagnostic markers for heart failure. SIRT6 (HDAC) is a protective factor against the development of cardiac hypertrophy, and a decrease in its expression has also been found in the onset of heart failure [77]. HDAC9 can be detected in advanced heart failure stages, and a higher cardiac function grade correlates with greater HDAC9 expression levels, indicating that HDAC9 is positively correlated with the severity of heart failure [78]. HDAC9 shows the best potential as an indicator of the severity of heart failure. HDAC4 dynamically adjusts the expression of cardiac ANP and BNP under conditions that can dynamically adjust cardiac preload and afterload, participating in the development of the disease [79]. Tamoxifen-induced double knockout of cardiomyocyte BRG1 and BRM genes in mice aged 3–6 months can lead to rapidly progressive heart failure, which is lethal [80]. The HAT P300 is involved in heart failure development. Inhibiting histone H3K9 acetylation when P300 is silenced promotes heart failure by cardiac hypertrophy, stimulation, stress load, and other factors [59]. The HDM KDM5B, which is involved in stress overload or myocardial infarction, is significantly upregulated under pathological stress induction. KDM5B knockout improves cardiac remodeling, myocardial fibrosis, and cardiac function [81].

**Fig. 2:**
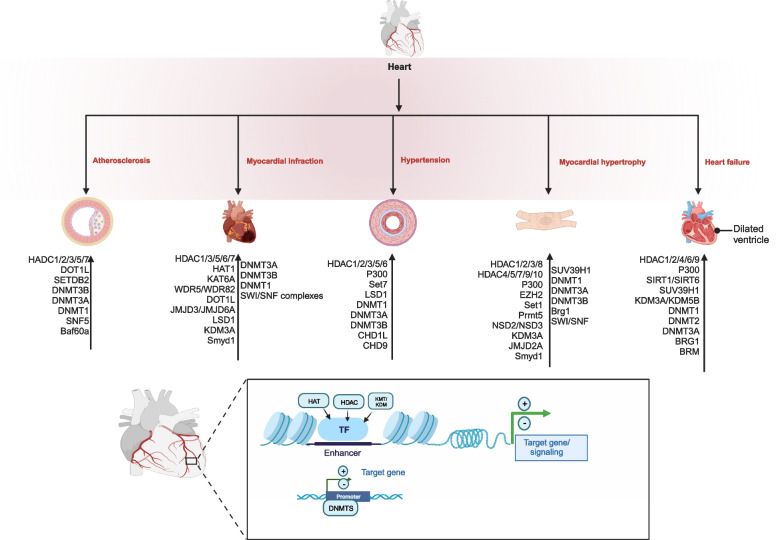
Chromatin-modifiers regulate transcription factors and signaling pathways involved in a variety of cardiovascular disease processes. The participation of chromatin modifiers was summarized according to the classification of diseases

Chromatin modifiers play a role in the end-state process of various cardiovascular diseases, enhancing or worsening cardiac insufficiency. Several studies [82, 83] have demonstrated somatic mutations in DNMT3A and TET2 in peripheral blood samples of patients with chronic ischemic heart failure, suggesting that they are associated with a poor prognostic outcome. HDAC1 and HDAC2 regulate ventricular arrhythmias in the early stages of heart failure. HDAC2 silencing affects the mRNA levels of calcium-activated potassium channels, resulting in delayed repolarization and prolonged action potentials [84]. Silencing of the HMT SUV39H1 can block the protective effect of sacubitril/valsartan on myocardial injury and heart failure after myocardial infarction [85]. SUV39H1 can bind to the promoter of the HDAC SIRT1 to inhibit its transcription of SIRT1, ultimately exacerbating the decline in cardiac function after ischemia–reperfusion injury. However, it can improve damaged heart lesions and heart function when silenced SUV39H1 [86]. Intestinal dysbacteriosis after myocardial infarction can affect HDAC activity by regulating butyric acid production in the intestine, thereby promoting the occurrence of cardiac insufficiency [87]. Cardioexcitatory contractile coupling requires the sarcoplasmic reticulum to supply and recycle calcium ions to assist in the contractile and diastolic movements of the heart. In rats with diabetes mellitus and myocardial infarction, downregulation of DNMT1 and DNMT3A inhibits the methylation of the inophosphine promoter, which in turn leads to impaired sarcoplasmic reticulum calcium uptake, decreased myocardial contractility, and worsened cardiac insufficiency [88]. In conclusion, differential regulation of chromatin modifiers with a single gene at different stages of heart failure is a good starting point for research (Fig. 2).

### Cancer

The pathogenesis of cancer is still not fully understood, making it a complex and multifactorial disease. Proto-oncogenes and tumor suppressor genes balance each other to maintain the relative stability of positive and negative regulatory signals. Proto-oncogenes transform normal cells into functional oncogenes in four ways: acquisition of strong promoters and enhancers, chromosomal translocations, gene amplification, and point mutations, all of which are inseparable from the regulation of chromatin modifiers. For example, crosstalk between histone-modifying enzymes and enhancers promotes the activation of proto-oncogenes, causing tumors such as liver, breast, and colorectal cancers [89–92]. The G protein-coupled receptor A2A activates the proto-oncogene MYC to promote proline synthesis, and the increased level of proline metabolism recruits histone acetylases SIRT6 and SIRT7 to mediate the level of H3 deacetylation in prostate cancer cells, triggering the transformation of prostate adenocarcinoma into a neuroendocrine tumor [93]. Similarly, P300 synergizes with BRD4 (transcriptional cofactor) to promote the activation of the proto-oncogene MYC in aggressive squamous cell carcinoma [94].

However, the high DNA methylation level of tumor suppressor genes or the dynamic changes in histone acetylation of tumor suppressor genes can lead to gene transcriptional repression, which disrupts the balance between proto-oncogenes and tumor suppressor genes. Krüppel-like factors are a highly conserved family of transcription factors closely related to multiclass tumorigenesis. First, Kleppel-like factor-9 (KLF9) can recruit HDAC1 to reduce the level of acetylation at the binding site of KLF9 to the matrix metallopeptidase 9 (MMP9) promoter, thereby inhibiting the invasion and migration of breast cancer cells [95]. Second, there is evidence that combining KLF9 with Pabicistat (LBH589), an HDAC inhibitor, can induce apoptosis in glioma stem cells and multiple myeloma cells [96, 97]. KLF4 exhibits high levels of DNA methylation in hepatocellular carcinoma, while HDAC3 inhibits KLF4 promoter activity in lung tumors [98, 99]. The above evidence suggests that members of the Krüppel-like Factors family, such as KLF9 and KLF4, are potent tumor suppressors. It also indicates that the same transcription factor is regulated by different chromatin modifiers in distinct diseases. The Krüppel-like Factors family is just one of the many tumor suppressors, and there are many more tumor suppressor genes are regulated by chromatin modifiers [100]. Uncovering the molecular mechanisms that explain the cancer-promoting or cancer-inhibiting properties of these chromatin modifiers can establish a critical foundation for precision medicine strategies to cancer treatment.

### Nervous system disease

The human brain has an extremely complex communication system - the nervous system, which not only receives a large number of signals but also sends out a large number of instructions. However, the nervous system is susceptible to damage from disease and trauma, such as cerebral endothelial injury and thrombosis due to inflammation. Nerve cells can degenerate, leading to Alzheimer’s disease or Parkinson’s disease. Bacteria or viruses can infect brain tissue or spinal cord, causing encephalitis or meningitis. Healthy neurons maintain normal function, while neurological diseases with poor prognosis pose a serious threat to human life and quality of life. Chromatin modifiers are closely related to neurodevelopment. Recently published studies have confirmed that patients with HMT SETD2 mutations present with intellectual disability, developmental delay, and skeletal system abnormalities as the main clinical manifestations [101]. However, mutations in the domain between HMT NSD1 exon 10 and 22 are associated with microcephaly [102]. The above evidence suggests that histone methyltransferases are closely associated with neurodevelopmental disorders.

Chromatin modifiers are also involved in regulating a person’s mood and memory. The plasma cholesterol metabolite 27-hydroxycholesterol can induce an increase in HDAC activity, leading to aggravated synaptic damage in rats with Alzheimer’s disease (AD), resulting in impaired memory [103]. Moreover, HDAC also affects the crosstalk between endoplasmic reticulum-mitochondrial organelles in the hippocampus of AD mice and aggravates the microglial inflammatory response in the entorhinal cortex, causing cognitive impairment [104, 105]. In addition, mice with conditional knockout of the HDAC9 gene from forebrain neurons reversed depressive disorders caused by chronic restraint stress [106]. In addition, HDAC11 is also involved in regulating the homeostasis of autophagy and nitric oxide in the microglia of depression-like mice [107].

Ginsenoside nanoparticles induce the expression of histone demethylases JMJD3 and UTX to increase the levels of demethylation in the promoter regions of VEGF-A and Jagged1, thereby promoting angiogenesis in rats with ischemic stroke [108]. Highly expressed DNMT1 and DNMT3A were detected in the spinal cord and skeletal muscle of mice with amyotrophic lateral sclerosis; however, treatment with the DNMT inhibitor RG108 improved motor function and prolonged survival in the affected mice [109].

### Immunological disease

The classification of immune diseases is based on disorders of immune regulation that impair the body’s immune function. Cytokine response, dysregulation of the inflammatory response, enzyme system defect, and T-cell dysfunction are the main mechanisms of immune function homeostasis and imbalance. The Tcf1, Gata3, Bcl11b, and Runx transcription factor families are major players in the T cell development-related transcriptional network. However, the aberrant regulation of chromatin modifiers in these members leads to T-cell development disorders and induces the occurrence of related diseases. For example, Tcf1 is both a transcription factor and a histone deacetylase (HDAC), and the HDAC activity of Tcf1 helps Ctla4 inhibit the function of follicular helper T cells, which has an important impact on the design of vaccines [110, 111]. Moreover, the long subtype of Tcf1 is associated with the pathogenesis of acute viral infection [112]. Histone methyltransferase mixed lineage leukemia 1 (Mll1) also regulates the expression of Tcf1 and promotes follicular helper T cell differentiation [113]. Both intranasal and systemic administration of the HDAC inhibitor Butyrate in patients with allergic airway inflammation may inhibit type 2 innate lymphocyte proliferation and IL-5 and IL-13 cytokine production by inhibiting GATA3 expression [114].

Studies have confirmed that the recruitment of monocytes and macrophages plays an important role in the development of immune diseases. By comparing multiple HDAC inhibitors, HDAC3 could inhibit the secretion of cytokines such as TNFα and IL-6 in monocytes and M1 macrophages induced by Lipopolysaccharides, and also reduced the tolerance of M1 macrophages to Lipopolysaccharides [115]. Sepsis can recruit a large number of monocyte exosomes to induce DNMT aggregation and induce immunosuppression [116]. Dysregulation of the inflammatory response is inseparable from the disrupting action of inflammasomes (complexes of platform proteins, adaptor proteins and effector proteins) [117]. Chromatin modifiers have a unique regulatory role in activating and silencing inflammasome receptor proteins. The results showed that the down-regulation of microRNA 145 expression detected in atherosclerotic plaques could induce NLRP3 inflammasome activation, and the decrease in microRNA 145 expression was associated with the enrichment of DNMT1 and the hypermethylation status of its promoter [118]. In addition, other studies have shown that HDAC6 and HDAC2 are also involved in the activation of NLRP3 inflammasomes in inflammatory diseases such as tuberculosis infection [119, 120]. Glycolic acid stimulation of human immortalized keratinocytes induces DNMT3B recruitment in NLRC4 inflammasome and inhibits the transcriptional expression of NLRC4 and ASC, which may lead to the blockage of NLRC4 inflammasome assembly [121].

## Types of chromatin modifiers

Chromatin changes dynamically in the growth and development of eukaryotes, so changes in the external environment or the internal environment of the body can cause mutations in gene loci and even induce diseases. There has been substantial research evidence that chromatin modifiers play an integral role in disease development. Therefore, more comprehensive elucidation is necessary to ascertain the diverse biological characteristics and functional roles of separate chromatin modifiers in illnesses. Chemical modifications of DNA and non-coding RNAs, post-translational modifications of histones, and higher-order chromatin structure are traditionally transcriptional modifications of gene expression. However, chromatin modifiers only regulate the degree of physical contact between cis-regulatory factor-transcription factor and chromatin DNA to change the size of the chromatin open area, further promoting or inhibiting transcription factor binding to gene enhancers and promoters. This regulation gives each cell type dynamic and specific gene expression characteristics. The mis-regulation of relevant epigenetic processes can be caused by changes in transcriptional regulation, and progressive, cumulative genomic changes always result in disease states. It is predicted that the intervention of abnormal chromatin modifier regulation will be beneficial for human health. Currently, chromatin modifiers are broadly classified into three groups: DNA methyltransferases, histone modifiers, and chromatin-remodeling complexes.

### Histone modifying enzymes

Histone-related modifications are classified mainly as “writers, erasers, readers”. Writers refer to enzymes that regulate histone modification to relax or tighten the chromatin after responding to external stimuli [122]. Histone-modifying enzymes have a dual role in regulating gene expression. On the one hand, they loosen the attachment of DNA to the nucleosome, which leads to the expansion of the open region of chromatin. It allows transcriptional regulatory programs to be activated. On the other hand, these enzymes can cause chromatin structure to condense, resulting in the shrinkage of the open region and blocking promoter entry, thereby hindering the process of transcriptional regulation. Histone-modifying enzymes include HATs, HDACs, HMTs, histone demethylases (HDMs), histone phosphorylases, and others [4]. Nucleosomes are in a dynamic state where the addition of acetyl groups to lysine residues by HATs can increase the level of acetylation between histones and linker DNA, enabling transcriptional activation and initiation of gene expression. Conversely, HDACs can remove histone residues to increase contact between nucleosomes, causing chromatin condensation and inhibiting transcriptional regulation. Both HATs and HDACs play important roles in modifying chromatin structure and cell fate.

HATs can divided into five categories according to their different substrate recognition modes, regulatory modes, and sequence homology: HAT1 (KAT1), Gcn5/PCAF (KAT2A/KAT2B) general control non-depressible 5, p300/CBP (KAT3B/KAT3A), MYST (KAT5) and KAT11 [123]. Various HATs play an important role in signal transduction pathways and molecular mechanisms such as cancer cell transformation, cell adhesion, DNA damage and repair, cell fate decision-making, and mitochondrial fatty acid oxidation, which makes it a key player in human diseases such as cancer, aging, neurological diseases, and cardiovascular diseases [124–126]. The corresponding enzyme of HAT is HDAC. It has been shown that HDACs are classified into 4 categories, namely Class I HDACs (HDAC1, 2, 3, 8), Class II HDACs (HDAC4, 5, 7, 9, 10), Class III Sirt Family-Nicotinamide Adenine Dinucleotide-Dependent Enzymes, and Class IV HDAC11-Zinc-Dependent Enzymes [127]. The huge HDAC family has a powerful functional role in regulating biological processes like cardiac gene reprogramming, tumor cell proliferation and migration, neuronal synaptic vesicle endocytosis, drug design, stem cell fate, apoptosis, etc., and has the ability to participate in the occurrence of multi-system diseases and become promising drug development targets [128–133].

Histone phosphorylases add negative charge to histones, facilitate the interaction of chromatin regulatory mechanisms with transcription factors, and adjust the subtle structure of chromatin, thereby influencing transcriptional regulation. It is closely related to biogenesis processes such as cell growth, regulation of AKT signaling pathway, chromosome condensation, DNA repair, mitosis, and programmed cell death. For example, the histone phosphorylase AURKA phosphorylates Ser10 of histone H3 in the S phase of mitosis and counteracts the production of R-loop in the treatment of MYCN-driven neuroblastoma [134]. Additionally, AURKA also connects to the regulation of DNA damage and cell cycle following radiation therapy [135, 136].

HMTs, which assist in histone methylation and modify histone arginine or lysine residues, play a role in maintaining chromatin status and are associated with DNA repair, mitosis, and cell-cycle regulation [137, 138]. The histone lysine methyltransferase family predominantly regulates histone H3 N-terminal tails and H4 N-terminal lysine residues, each of which undergoes methyl transfer but forms a trimethylated derivative at most [139]. The protein arginine methyltransferase family (PRMTs) catalyzes the formation of monomethylarginine and symmetric or asymmetric dimethylarginine by using transfer methyl groups to replace potential hydrogen bond donors in arginine residues [140]. In contrast, HDM exerts a demethylating function to be primarily involved in the transcriptional regulation and expression of activation-related genes [38]. Currently, HDM is mainly composed of flavin adenine dinucleotide-dependent lysine demethylase (KDM1) and a demethylase (KDM2-7) containing α-ketoglutarate or iron(II)-dependent jumonji-C domain. The balance of HMT and HDM activities allows for fine transcriptional regulation in normal cells, but when this equilibrium is broken, transcriptional regulation is hampered. For example, KDM5B is associated with the development of hypertension and is involved in myocardial fibrosis and remodeling [141]. KDM5B can also be involved in the immune escape mechanism of melanoma in combination with HMT SETDB1 [142]. PRMT5 is elevated in various hematologic malignancies and solid cancers and is responsible for maintaining muscle development and regeneration under normal physiology [143–146]. However, PRMT5 has more than just a promoting effect in cancer, and it has been confirmed that PRMT5 has a tumor-suppressive effect in clear cell renal cell carcinoma [147].

### DNA methylases

DNMTs regulate DNA methylation and are involved in transcriptional regulation and gene expression. DNMTs assist in DNA methylation by transferring the methyl group of S-adenosylmethionine to the cytosine base via covalent modification. This regulatory activity is located mostly in the promoters of short genomes rich in CpG islands, inhibiting chromatin and gene expression. The DNMT family is divided into five categories: 1, 2, 3A, 3B, and 3L [28]. Studies have confirmed that the UHRF protein family, zinc finger protein family, and methyl CPG binding domain family can recognize DNA methylation and bind methyl groups [148] DNA demethylases help modify methyl groups and remove methylation. Although no definitive DNA demethylases have been identified, studies have shown that demethylation is achieved by TET enzymes [149]. Different protein families play different transcriptional regulatory roles via methylation. For example, DNMTs have been reported to inhibit the expression of FGFR1 to promote metabolic disorders in the body, thereby damaging cardiomyocytes and disrupting cardiac mitochondrial function [150]. In the programmed death of pulmonary artery endothelial cells, new pathophysiological evidence for hypoxic pulmonary hypertension shows that FENDRR inhibits the DNA methylation of DRP1 to inhibit cell pyroptosis. However, n6-methyladenosine (m6A) modification and editing can reverse this phenomenon and increase pyroptosis in pulmonary artery endothelial cells [151]. Under the interaction of DNMT1 and HMT G9a, homocysteine inhibits the expression of reticulo-oxidoreductase 1α (ERO1α), activates the occurrence of endoplasmic reticulum stress and hepatocyte apoptosis, causing liver injury [152]. In addition, DNA methylases are also involved in the progression of chronic kidney disease to renal cell carcinoma [153].

### ATPase-dependent chromatin-remodeling complexes

ATPase-dependent chromatin-remodeling complexes utilize ATPases for ATP hydrolysis. The energy released by ATP is used to disrupt histone–DNA interactions. Because local or global chromatin structures are subjected to non-covalent regulation, this allows the dynamic sliding or repositioning of nucleosomes to recruit transcriptional coactivators and corepressors to DNA promoters for epigenetic regulation [154, 155]. In addition, ATPase-dependent chromatin-remodeling complexes include INO80, ACF, SWI/SNF, chromodomain helicase DNA-binding (CHD), and ISWI, which are associated with DNA repair, indicating a link between repair and remodeling activity. Notably, histones are not covalently altered by the ATPase-dependent chromatin-remodeling complex, which functions via DNA-accessible regulation [156, 157]. ATPase-dependent chromatin-remodeling complexes are associated with the occurrence and development of coronary heart disease, heart failure, pathological myocardial hypertrophy, and other cardiovascular diseases. Although there are few studies on the regulation of ATP-dependent nucleosome chromatin remodeling complexes in cardiovascular diseases, they are rich in research fields such as malignant tumors, lipid metabolism, immune regulation, DNA damage and repair, and autophagy [158–161]. For example, the SWI/SNF complex contains multiple subunits, and different subunits drive the occurrence of different diseases. AT-interacting domain-rich protein 1A (ARID1A) is one of the SWI/SNF complex subunits that control the development and regeneration of the bladder urothelium. ARID1A mutation leads to self-inactivation but induces the occurrence of bladder tumors and affects the immune invasion process of bladder cancer, and ARID1A mutation is associated with poor prognosis [162–164]. Moreover, the deletion of ARID1A in the tumor microenvironment activates cancer-associated fibroblasts and drives the proliferation and migration of lung cancer cells, but inhibits tumor cell autophagy and enhances the sensitivity of immunosuppressive therapy for EGFR-mutant lung adenocarcinoma [165, 166].

## Regulatory mechanisms of chromatin modifiers

**Fig. 3:**
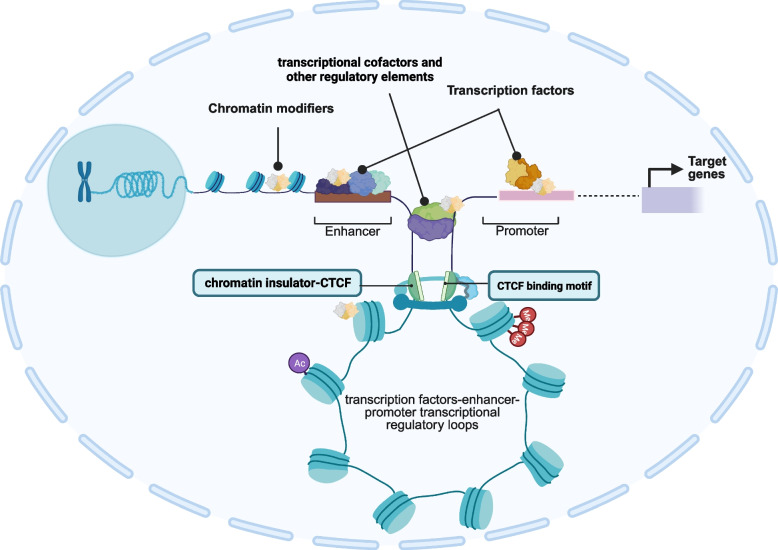
A panorama of chromatin modifiers involved in the formation of transcriptional regulatory loops. Chromatin modifiers work with enhancers, upstream promoter elements, enhancers, insulators (CTCFs), transcription factors, transcription cofactors, and other transcriptional regulatory elements to regulate transcriptional regulatory programs

Chromatin modifiers are a class of trans-acting factors that can bind to cis-acting elements such as promoters, upstream promoter elements, enhancers, and insulators localized in open chromatin regions. This binding plays a key role in regulating transcriptional efficiency or transcriptional repression of genes. It is important to note that chromatin modifiers do not code DNA sequences for RNA and proteins (Fig. 3). Chromatin modifiers are also part of the epigenetic regulators related to chromatin accessibility, which affect the modified transcriptional regulatory circuit and the post-transcriptional regulation and protein translation process. Summarizing the modification mechanism of chromatin modifiers to key loci in the transcriptional regulatory circuit is more conducive to understanding their crucial position in the transcriptional link of regulatory genes.

Firstly, chromatin modifiers are related to the activity of enhancers. In cis-acting elements, the active enhancer already has two typical recognizable histone markers: histone H3 lysine 27 acetylation (H3K27ac) and histone H3 lysine 4 monomethylation (H3K4me1) [167]. The activation of enhancers in transcriptional regulation is a process involving the participation of multiple chromatin modifiers, including MLL3 (KMT2C) and MLL4 (KMT2D) assisting each other to produce monomethylation of H3K4, followed by recruitment of H3K4me1 to stimulate p300/CBP to increase H3K27ac levels [168]. DNA methyltransferases may regulate high levels of methylation associated with transcriptional silencing. For example, enhancer activity increased using PRO-seq sequencing in Dnmt1, Dnmt3a, and Dnmt3b triple-knockout mice, while enhancer activity decreased after the same measurement after TET knockout [169].

**Fig. 4:**
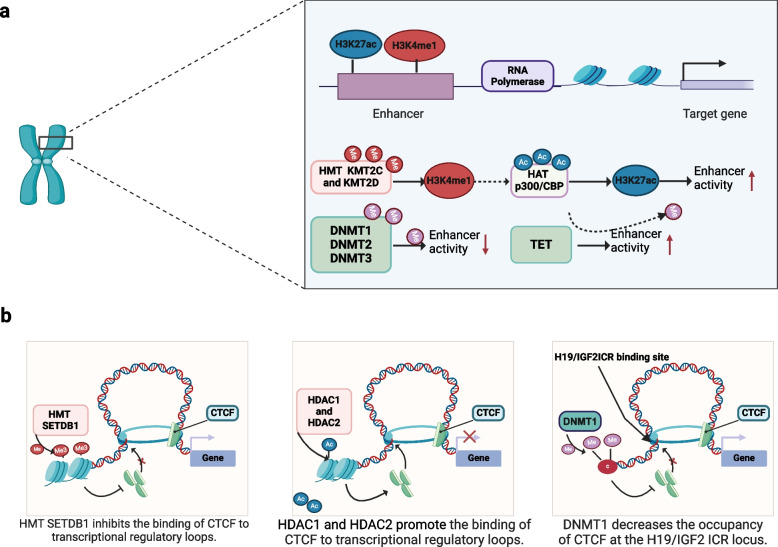
Chromatin modifiers act in concert with super-enhancers, or CTCF, to coordinate the regulation of gene transcriptional expression.** a** In terms of chromatin modifier and enhancer collaboration, KMT2C and KMT2 work together to increase H3K4me1, which helps to attract p300/CBP and stimulate H3K27 production. This process activates enhancers and promotes transcription program initiation. However, enhancer activity can be inhibited by DNMT1/2/3. The inhibitory effect of DNMT can be reversed by TET, which helps to promote enhancer activity. **b** The cooperation between chromatin modifiers and insulator CTCF affects gene expression. HMT SETDB1 helps loose the chromatin structure by increasing H3K9me3 and hindering CTCF from binding to transcriptional regulatory loops, thus activating transcription. On the other hand, HDAC1 and HDAC2 assist CTCF in binding to transcriptional regulatory loops, which keeps the chromatin structure compact and inhibits transcription. DNMT1 promotes hypermethylation at H19ICR/IGF2, which prevents CTCF from binding to H19ICR/IGF2 and inhibits gene expression

Secondly, chromatin modifiers and CTCF interact with each other to maintain the stability of the transcriptional circuit. The chromatin insulator has the ability to block long-range communication between enhancers and promoters in the transcriptional regulatory loop, inhibiting the recruitment of related regulatory elements [170]. CTCF, which consists of an N-terminal domain and 11 zinc finger domains, has been found to be involved in the regulation of chromatin accessibility by chromatin modifiers. Abnormalities in chromatin modifiers have also been identified as one of the factors responsible for the dysfunction of CTCF. In HMT SETDB1-deleted embryonic stem cells, the deletion of H3K9me3 caused the chromatin structure to become compact, which further promoted the increased binding of CTCF to the transcriptional regulatory loop and inhibited transcriptional regulation [171]. Endometrial adenocarcinoma cells and human cervical cancer cells have been found to contain HDAC1 and HDAC2, which help in the binding of CTCF to DNA elements, maintain compactness of chromatin structure, and inhibit transcription. However, when HDAC function is inhibited, histone acetylation reverses the occupancy of CTCF and promotes chromatin structure relaxation to activate transcription [172]. In addition, the observed increase in histone acetylation levels in human non-small cell lung cancer cells and human lymphoma cells can promote the expression of DNMT1, cause hypermethylation at the ICR binding site of CTCF-H19/IGF2, and reduce the occupancy of CTCF at the ICR of H19/IGF2 [173]. Similarly, the SWI/SNF remodeling complex has been found to be a chaperone for CTCF, which binds to the central DNA-binding domain of the zinc finger [174]. In summary, CTCF and chromatin modifiers are synergistic or antagonistic to maintain chromatin structure and affect gene regulation. The intrinsic sequence structure characteristics contained in cis-acting elements often directly determine the binding and mode of action of chromatin modifiers, and the differential regulation of chromatin modifiers in different biological processes can also affect the activation of cis-acting elements (Fig. 4).

Furthermore, chromatin modifiers and long non-coding RNAs (lncRNAs) regulate each other. lncRNAs have a crucial role in the regulation of gene expression, both in transcriptional and post-transcriptional regulation. Moreover, they have an impact on the regulatory function of chromatin modifiers. For instance, EZH2 is a target for many noncoding RNAs involved in the development of heart failure. The lncRNA NEAT1 recruits EZH2 to the Smad7 promoter, increases Smad7 methylation, and accelerates the development of heart failure and ventricular remodeling [175]. Also, EZH2 can promote the methylation level of mir-34a to inhibit the apoptosis of cardiomyocytes after acute myocardial infarction and improve myocardial injury [176]. In addition, lncRNA ANRIL is upregulated in patients with coronary atherosclerotic heart disease and promotes the binding of WDR5 (the key subunit of the HMT H3K4MT complex) to HDAC3 to form the WRD5–HDAC3 complex, which regulates oxidative stress and increases reactive oxygen species (ROS) levels [177]. This phenomenon confirms that lncRNAs can also direct synergistic regulation between chromatin modifiers.

Finally, chromatin modifiers are functionally cooperative and independent of each other. Cross-intercommunication between DNA methyltransferases and histone modifiers has been demonstrated in a variety of diseases like hypertensive disorders, periodontitis, epilepsy, and cancer [178–183]. In quiescent cells, monophosphorylation at nucleosome histone 3 (H3) and phosphoacetylation increased upon activation of proto-oncogene c-Jun. H3 acetylation was not inhibited after treatment with inhibition of H3 phosphorylation, and H3 acetylation was significantly up-regulated after HDAC inhibitor treatment, but the level of phosphoacetylation did not change [184]. In addition, it was also observed that H3 was first phosphorylated and then rapidly acetylated to produce H3 phosphoacetylation in response to epidermal growth factor and methyl acetyl phosphate [185, 186].

In this way, the parallel independent and synergistic coupling connection between histone acetylation and phosphorylation was confirmed. In 2003, Fischle et al. discovered that the phosphorylation of histone H3 serine 10 (H3S10ph) in the mitotic interphase interferes with the binding of chromatin protein 1 (HP1) to the adjacent H3K9me3 to maintain the regular operation of the cell cycle, and defined this phenomenon as the binary switch hypothesis [187]. In the binary switch hypothesis, also known as the binary methylation-phosphorylation switch, Aurora B and SUV39H1 have been identified to maintain the kinetic equilibrium of heterochromatin [188]. Subsequently, the acetylation level of H3K14 was enhanced by the confirmation of a crosstalk relationship between histone phosphorylase and histone methyltransferase in Arabidopsis thaliana cells, whereas in the opposite case, the prior increase in H3K14ac level did not promote phosphorylation of H3S10 [189]. Thus, the tertiary cyclic relationship between the three histone modifications was revealed.

## Emerging therapeutic strategies targeting chromatin modifiers

There is mounting significant evidence to comprehend the diverse molecular pathways and mechanisms that cause diseases due to disorders in the role of chromatin modifiers. Targeting the function and regulation of chromatin modifiers can facilitate the development of gene therapies aimed at these targets. Currently, the WHO international clinical trial registry platform has 853 clinical research projects related to HDAC inhibitors, 21 clinical research projects of EZH2, and 32 clinical research projects about DNMT inhibitors. More than 1,000 articles are summarized about the therapy of chromatin modifier inhibitors, especially for cancer, hematologic malignancies, and immunotherapy. It is clear from these data that the field is gradually gaining interest from researchers [190].

Chromatin modifiers provide greater targeting characteristics and pharmacokinetics, less toxicity and side effects, and easier treatment options in the treatment prospect of chemical agents. For example, the treatment of ejection fraction-preserving heart failure with suberoylanilide (class I and II HDAC inhibitors) increased cardiomyocyte contractility, calcium processing rate, myofilament calcium sensitivity, and other properties, and improved mean pulmonary artery pressure and left ventricular end–diastolic pressure [191, 192]. Another class I HDAC selective inhibitor, sodium valproate, is a new oral controlled-release formulation, CS1, which is currently in phase 2 clinical trials. The results of phase 1 and phase 2 clinical trials demonstrate that CS1 exhibits efficacy with a significant reduction in plasminogen levels, improvement of coagulation function, and decreased risk of bleeding in patients diagnosed with pulmonary hypertension. Good safety and tolerability are observed alongside these effects [193]. Moreover, CS1 is not restricted by the maximum tolerated dose or dose-limiting toxicity. DNMT inhibitors offer significant advantages over conventional treatments for related malignancies. Not only do they prolong median survival, but they also have negligible toxicity and side effects. When combined with carboplatin or nab-paclitaxel in the treatment of nasopharyngeal carcinoma, DNMT inhibitors are effective in reducing the occurrence of bone marrow suppression [190]. Their proven efficacy and safety make DNMT inhibitors a promising option for those seeking improved outcomes and reduced side effects in cancer treatment. The newly discovered irreversible HDAC6 inhibitor, phenylsulfonylfuroxan-based hydroxamate 4, has superior anti-multiple myeloma activity compared to ACY-241, an HDAC6 inhibitor in clinical trials [194].

Certain medications used in clinical practice have been observed to target chromatin modifiers and exhibit potential anti-cancer effects. A case in point is the anti-heart failure drug, sacubitrilat, which used to trigger apoptosis in colon and breast cancer cells by significantly downregulating the level of histone deacetylases (HDACs) [195]. This discovery highlights the potential to repurpose drugs to combat cancer. The hypoglycemic drug metformin has been found to partially impede the migration and invasion of prostate cancer cells by inhibiting HMT SUV39H1 [196]. Furthermore, it has been discovered that metformin can also hinder the proliferation and migration of ovarian cancer cells by inhibiting HMT EZH2 [197]. Then, metformin has been shown to reverse the insensitivity of multidrug-resistant breast cancer to chemotherapy drugs such as DOX through the upregulation of histone acetylation [198]. These findings suggest that metformin may have potential therapeutic applications in the treatment of cancer, particularly in the context of drug resistance. The development of dual inhibitors targeting phosphoinositide 3-kinase (PI3K) and HDAC for the treatment of acute myeloid leukemia (AML) has been confirmed. These inhibitors have been rigorously designed, and their efficacy has been demonstrated to surpass that of their respective single-target counterparts, owing to their superior metabolic stability. The discovery of such inhibitors is a promising advancement in the treatment of AML [199]. Medical research continues to make noteworthy strides in this regard, and it is hoped that further exploration in this area will lead to the development of more effective cancer treatments.

In the investigation of traditional Chinese medicine and natural medicine treatment methodologies, it has been discovered that various plant extracts possess inhibitors of chromatin modifiers. An extract from a newly discovered brown alga growing in the Sea of Japan, Ecklonia stolonifera Okamura extract (ESE), can be used to treat phenylephrine-stimulated rat primary cardiomyocytes, inhibiting histone acetylation levels, P300 activity, and transcription of hypertrophy-related genes [200]. This finding suggests that ESE inhibits cardiomyocyte hypertrophy by decreasing the level of histone acetylation regulated by P300. The classical inhibitor of P300 curcumin has been used in the treatment of cardiovascular diseases, cancer, skin diseases, aging, nervous system, immune abnormalities and other diseases. The anti-cancer and anti-inflammatory effects of curcumin are too numerous to enumerate. Curcumin has also been effective in lowering blood pressure, improving cardiac function, and inhibiting ventricular remodeling in cardiovascular diseases. For instance, the P300 inhibitor curcumin inhibits hypertension-induced heart failure and post-myocardial infarction heart failure by preventing left ventricular systolic dysfunction in the heart failure stage [201, 202]. The anthraquinone emodin, extracted from plants, improves pathological myocardial hypertrophy in patients with heart failure by inhibiting HDAC activity [203]. In addition, studies have shown that curcumin inhibits the acetylation level of upstream genes, thereby inhibiting the activation of myocardial hypertrophy and fibrosis-related genes, reducing the thickness of the posterior wall of the heart and inhibiting left ventricular hypertrophy in hypertensive rats [201]. Moreover, another inhibitor of P300, L002, has been identified as having the efficacy of alleviating myocardial fibrosis after hypertension [204, 205].

The discovery and identification of new strategic points for drug development and treatment of various diseases has been a topic of interest in recent years. In this regard, Gallic acid, Resveratrol, Clinacanthus nutan, Andrographolide, ligistin, Wogonin, and Triptolide have been identified as potential candidates for inhibiting their target chromatin modifiers [206–212]. The identification of these inhibitors in plant extracts presents an intriguing avenue for further exploration and development of novel therapies. Further research is necessary to determine the efficacy and safety of these plant extracts, and to understand their mechanism of action in inhibiting chromatin modifiers. Nonetheless, this discovery offers a promising direction for the development of new treatments based on natural compounds. Givinoistat, a pan-histone deacetylase inhibitor (HDACi), has demonstrated safety and efficacy in humans [213, 214]. This compound exhibits anti-inflammatory and immunoregulatory activity against several inflammatory diseases, type 1 diabetes and traumatic brain injury [215–218]. Furthermore, Givinoistat has demonstrated anti-tumor activity against hematologic malignancies and solid tumor cancers by inducing apoptosis [219–222]. It should be noted that Givinoistat has displayed a protective impact in managing cardiovascular diseases, as suggested by recent research studies. It relieves myocardial fibrosis, myocardial decompensated hypertrophy, and promotes angiogenesis, while exhibiting a cardioprotective effect [223]. However, Givinoistat has been observed to inhibit cardiomyocyte apoptosis in myocardial infarction, contrary to its pro-apoptotic effect in tumors. Additionally, Givinoistat has been found to have protective effects on left ventricular diastolic function and cardiac remodeling in hypertension and aging, highlighting its potential as an anti-heart failure drug target [224, 225].

**Fig. 5:**
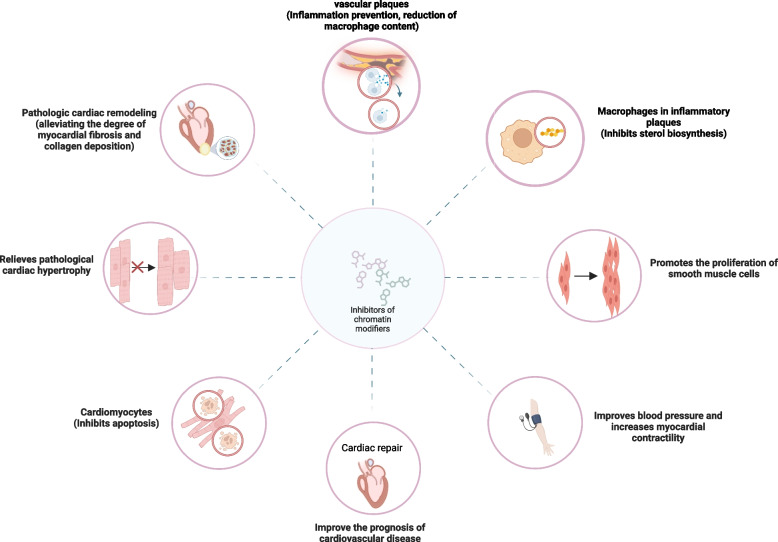
Therapeutic effects of chromatin inhibitors in cardiovascular disease. Chromatin modifier inhibitors can improve vascular function, prevent inflammation in vascular plaques, protect against the normal proliferation of smooth muscle cells, and reduce the risk of serious complications. It can also help in maintaining normal blood pressure and cardiac output, improving myocardial contractility, and inhibiting cardiomyocyte apoptosis. Additionally, it can alleviate myocardial fibrosis and collagen deposition, pathological cardiac hypertrophy, and remodeling, thereby improving the prognosis of cardiovascular diseases

Finally, the combination of chromatin modifiers and vaccine biologics has also been proposed to not only increase the titer of vaccines, but also increase safety and immunogenicity. The protein vaccine system (OVA + CpG) designed for melanoma not only prevents tumor formation, but also improves survival benefit after combining romidesine (HDAC inhibitor)-IBET151 (BET inhibitor) [226]. The DNMT inhibitor decitabine increases the immune effect of the NY-ESO-1 vaccine in ovarian cancer [227]. The treatment of HER2 breast cancer is currently being explored through a phase 1b clinical trial, which incorporates an innovative combination of the BN-Brachyury vaccine, bifunctional antibody protein, class I histone deacetylase inhibitors, and targeted drugs. The quadruple drug has demonstrated a stronger T cell immune response, as demonstrated by in vivo trials in mice. However, the safety and efficacy of this treatment are currently under evaluation. The results of this trial will provide valuable insights into the potential of this treatment for HER2 breast cancer [228]. In conclusion, with the development of therapeutic approaches that can normalize chromatin structure changes in gene expression, we have the potential to revolutionize clinical practice. By utilizing these approaches, we can unlock new pathways for treatment and improve patient outcomes. It is time to take advantage of this exciting field of research and explore the possibilities chromatin modifiers can offer. Figure 2 and Table 1 summarize the roles and effects of major chromatin modifiers and their inhibitors in cardiovascular disease (Fig. 5 and Table 1).

**Table 1:** Inhibitors of chromatin modifiers in cardiovascular dieasese associated with transcription

Inhibitors	Chromatin modifiers	Know effcts on TFs	Know roles in cardiovascular	Animals	References
SGC0946(EPZ-5676)	DOT1L	Inhibits Srebf1 and Srebf2	Inhibits sterol biosynthesis, promotes atherosclerotic plaque stability	Atherosclerosis mouse model	[14]
Valproic acid	class I/IIa-specific HDAC	enhanced the expression of Pdgfd and Sox12	Promotes repair function after heart injury	Mice with diabetic myocardial infarction	[27]
		enhanced the expression of Foxm1	Reduces infarct area and suppresses inflammatory reactions, improves cardiac function and metabolic expression programs, prevent CM apoptosis	MI rats	[30]
Metformin	P300	Inhibits ANF and BNP	Relieves myocardial hypertrophy	Neonatal rat cardiomyocytes	[59]
GO-Y030	P300	Inhibits GATA4	Relieves myocardial hypertrophy	Mice undergoing TAC surgery	[94]
suberoylanilide	Class I and II HDAC inhibitors	Upregulated Tfam, Pgc-1α, Pgc-1β, and Idh3α	Reduces left ventricular end-diastolic and mean pulmonary artery pressure, Improves ventricular remodeling and cardiac function	Feline model undergoing ischemia/reperfusion surgery and domestic short-hair felines undergoing TAC surgery	[191, 192]
Ecklonia stolonifera Okamura extract	P300	Inhibits ANF, BNP, α-SMA, and Collagen 1	Relieves myocardial hypertrophy	neonatal rat cardiomyocytes and MI rats	[200]
Curcumin	P300	Inhibits GATA4, serum responsive factor, and myocyte enhancer factor 2	Reduces the thickness of the posterior wall of the heart with hypertension and inhibits the progression of left ventricular hypertrophy, Inhibits myocardial fibrosis	Dahl salt-sensitive rats	[201]
Emodin	pan-HDAC inhibitor	Inhibits ANF, MAPK, and ERK	Improves pathological myocardial hypertrophy due to heart failure	Angiotensin II mouse model	[203]
L002	P300	Inhibits pSmad2, pERK1/2, and TGF-β signaling	Improves myocardial fibrosis levels due to hypertension	hypertensive cardio-renal fibrosis mice	[204]
Gallic acid	P300	Inhibits CaMKII, ANP, and BNP	Relieves hypertension by alleviating P53-dependent apoptosis	spontaneously hypertensive rats	[206]
Givinostat (ITF2357)	pan-HDAC inhibitor	Inhibition of gene expression associated with EndMT mechanism	Relieves myocardial fibrosis and inflammation after myocardial infarction, and improve cardiac function	AMI mouse	[223]
		-	Improves diastolic function in heart failure with preserved ejection fraction	Dahl salt-sensitive rats	[224]
		Inhibits the expression of genes associated with cardiac fibroblast activation	Improves diastolic function and relieves left ventricular sclerosis	Uninephrectomy and deoxycorticosterone acetate-salt hypertensive mouse	[225]
Valproic acid	class I/IIa-specific HDAC	Inhibits IbΔC-X and RhoA	Reduces the formation of blood clots in the atrial wall, slowing atrial remodeling, Delay the onset of atrial fibrillation and apoptosis of cardiomyocytes	Mice with cardiomyocyte-specific expression of CREM-IbΔC-X	[229]
Riboflavin	Activate LSD1	Inhibits Lpcat2 and Pld1	Protect the heart	MI mouse	[230]
LMK235	HDAC6	Inhibits CaMKIIα	Lowering systolic blood pressure, Reduces the proliferation of vascular smooth muscle	Spontaneously hypertensive rats	[231]
Curcumin	P300	Inhibits the binding of NFkB to inflammasome NLRP3	Reduces the process of inflammation of blood vessels and pathological remodeling of blood vessels, reduces the degree of hypertension	spontaneously hypertensive rats	[232]
Anacardic acid	HATs	Inhibits MEF2A, ANP, BNP, β-MHC, and JNK/MAPK signaling pathway	Relieves pathological cardiac hypertrophy	PE-induced mouse cardiomyocyte hypertrophy model	[233]
JS28	HDAC6	Inhibit Endothelin-1	Relieves heart failure	Puromycin-resistant male transgenic mouse embryonic stem cells	[234]
Teneligliptin	HDAC4	Inhibit the NOX4-HDAC4 axis	Relieves pathological cardiac hypertrophy	Ang II-induced model of mouse cardiac hypertrophy	[235]
RGFP966	HDAC3	Inhibits GATA6 and E2F3	Relieves the thickening of the walls of blood vessels and the occurrence of inflammation, Improve the prognosis of hypertension	Hypertensive mice	[236]
MS-275	Selective inhibitors of HDAC1, 2, 3	Inhibits GATA6 and E2F3	Relieves the thickening of the walls of blood vessels and the occurrence of inflammation, Improve the prognosis of hypertension	Hypertensive mice	[236]
Sodium valproate	HDACs	Inhibits ANP, BNP, Fibronectin, and Collagen-1/3	Relieves pathological cardiac hypertrophy	Rat undergoing TAC surgery	[237]
Mocetinostat	class-I HDACs	Activates the CREB/PGC-1α pathway	Relieves myocardial I/R injury, Protects the heart and kidneys	Rat undergoing TAC surgery, Ischemia/reperfusion model	[238, 239]
Romidepsin	HDAC1 and 2	Inhibits STAT3 acetylation and promotes DNMT1-STAT3 interaction, inhibits GATA6 expression	Reduces the development of atherosclerotic lesions	Apoe mice fed with a high-fat diet	[240]

## Summary and perspective

Research on chromatin modifiers has undergone a paradigm shift, moving from functional exploration to the involvement of multiple types of disease regulatory mechanisms. This transition underscores the substantial contribution of chromatin modifiers to our understanding of the pathogenesis of human disease. These findings suggest that chromatin modifiers play a crucial role in regulating gene expression, and their dysregulation may lead to various disease states. Furthermore, they have established the groundwork for creating novel therapeutic approaches targeting chromatin modifiers, which have the potential to treat a diverse range of diseases. Chromatin is loosely bound to the chromatin structural proteins and remains hyperdynamically active [241]. During cell differentiation, accompanied by chromatin modification and remodeling, chromatin transitions from a CG-rich open state to a compact and inhibitory state involving H3K27me3 and high DNA methylation, which increases the possibility of later epigenetic event initiation [241, 242]. Genetic omics-related transcriptional regulatory circuits are complex, diverse, profound, and publicly available. Abnormalities in transregulatory elements can even cause genomic regulation to become out of control.

As discussed in the text, chromatin modifiers have the potential as diagnostic targets, biomarkers, and disease-prognosis prediction factors in drug development and medical phenomena. Epigenetic changes are acquired through gradual accumulation. Epigenetic modifications and the assisting effects of chromatin modifiers are reversible. Therefore, because of their reversible characteristics, therapeutic strategies can be developed to restore the normalization of changes in gene expression and chromatin structure. In addition, multiple pieces of evidence suggest that the efficacy of chromatin modifier inhibitors is not limited to a single disease. For instance, Givinostat has anti-inflammatory, anti-immune, anti-tumor effects, and cardioprotective abilities. It has been found that drugs designed for chromatin modifiers can be used in combination to enhance their effectiveness in treating human diseases. For example, a combination of HDAC SIRT6 agonists and DNA methylase DNMT inhibitors can be used in treatment regimens for coronary heart disease to improve vascular conditions in coronary heart disease [243]. Moreover, epigenetic changes can be transmitted to the offspring, increasing the likelihood that the offspring of obese pregnant women will develop diabetes. Studies have shown that increased exercise in pregnant women can promote the expression of placental superoxide dismutase 3 to stabilize the promoter of glucose metabolism regulated by histone H3K4me3 levels; thus, restoring glucose metabolism disorders in offspring is possible [244]. The current detection of chromatin modifiers is extensive, and CTCF knockout technology can help monitor gene expression and chromatin structure–induced phenotypic changes during growth and disease development and check the integrity of modifications and chromatin accessibility [245].

In conclusion, histone-modifying enzymes and DNMT are involved in regulating chromatin structure, which in turn affects gene transcription and translation. The complex interaction between these elements has significant implications for gene expression and the overall functioning of a cell or organism. DNMTs participate in the regulation of chromatin structure, which in turn affects DNA transcription and translation. The body enters a disease state when DNA and histone post-translational modifications are abnormalized. Chromatin modifiers are associated with chromatin remodeling, providing energy by hydrolyzing ATP to disrupt the interaction between DNA and histones in nucleosomes, thereby changing the composition and structure of chromatin. In addition, correlations exist between chromatin modifiers. The parallel independent and synergistic coupling model between histone acetylation and phosphorylation, the binary switch hypothesis between histone methylation and phosphorylation, and the tertiary loop relationship between these three histone modifications make epigenetic regulation profound, complex, and exploratory.

## Data Availability

Not applicable.

## References

[1] Hogg SJ, Beavis PA, Dawson MA, Johnstone RW (2020). Targeting the epigenetic regulation of antitumour immunity. Nat Rev Drug Discov.

[2] Zhao Z, Aoi Y, Philips CN, Meghani KA, Gold SR, Yu Y (2023). Somatic mutations of MLL4/COMPASS induce cytoplasmic localization providing molecular insight into cancer prognosis and treatment. Proc Natl Acad Sci U S A.

[3] Han P, Hang CT, Yang J, Chang CP (2011). Chromatin remodeling in cardiovascular development and physiology. Circ Res.

[4] Allis CD, Jenuwein T (2016). The molecular hallmarks of epigenetic control. Nat Rev Genet.

[5] Sundaram MK, Unni S, Somvanshi P, Bhardwaj T, Mandal RK, Hussain A (2019). Genistein modulates signaling pathways and targets several epigenetic markers in HeLa cells. Genes (Basel).

[6] Wang F, Bai X, Wang Y, Jiang Y, Ai B, Zhang Y (2021). ATACdb: a comprehensive human chromatin accessibility database. Nucleic Acids Res.

[7] Qin XF, Shan YG, Dou M, Li FX, Guo YX (2023). Notch1 signaling activation alleviates coronary microvascular dysfunction through histone modification of Nrg-1 via the interaction between NICD and GCN5. Apoptosis.

[8] Lizcano F, Bustamante L (2022). Molecular perspectives in hypertrophic heart disease: an epigenetic approach from chromatin modification. Front Cell Dev Biol.

[9] Zhang T, Künne C, Ding D, Günther S, Guo X, Zhou Y (2022). Replication collisions induced by de-repressed S-phase transcription are connected with malignant transformation of adult stem cells. Nat Commun.

[10] Zhou P, VanDusen NJ, Zhang Y, Cao Y, Sethi I, Hu R (2023). Dynamic changes in P300 enhancers and enhancer-promoter contacts control mouse cardiomyocyte maturation. Dev Cell.

[11] Fan M, Yang K, Wang X, Chen L, Gill PS, Ha T (2023). Lactate promotes endothelial-to-mesenchymal transition via Snail1 lactylation after myocardial infarction. Sci Adv.

[12] Kawakami-Mori F, Nishimoto M, Reheman L, Kawarazaki W, Ayuzawa N, Ueda K (2018). Aberrant DNA methylation of hypothalamic angiotensin receptor in prenatal programmed hypertension. JCI Insight.

[13] Zhu L, Jia L, Liu N, Wu R, Guan G, Hui R (2022). DNA methyltransferase 3b accelerates the process of atherosclerosis. Oxid Med Cell Longev.

[14] Willemsen L, Prange KHM, Neele AE, van Roomen C, Gijbels M, Griffith GR (2022). DOT1L regulates lipid biosynthesis and inflammatory responses in macrophages and promotes atherosclerotic plaque stability. Cell Rep.

[15] Li S, Liu C, Li N, Hao T, Han T, Hill DE (2008). Genome-wide coactivation analysis of PGC-1alpha identifies BAF60a as a regulator of hepatic lipid metabolism. Cell Metab.

[16] Meng ZX, Wang L, Chang L, Sun J, Bao J, Li Y (2015). A diet-sensitive BAF60a-mediated pathway links hepatic bile acid metabolism to cholesterol absorption and atherosclerosis. Cell Rep.

[17] Chang SF, Tsai HE, Kuo JT, Ruan YR, Chen CY, Wang SY (2022). Blood reflux-induced epigenetic factors HDACs and DNMTs are associated with the development of human chronic venous disease. Int J Mol Sci.

[18] Lee DY, Lin TE, Lee CI, Zhou J, Huang YH, Lee PL (2017). MicroRNA-10a is crucial for endothelial response to different flow patterns via interaction of retinoid acid receptors and histone deacetylases. Proc Natl Acad Sci U S A.

[19] Subramanian V, Golledge J, Heywood EB, Bruemmer D, Daugherty A (2012). Regulation of peroxisome proliferator-activated receptor-gamma by angiotensin II via transforming growth factor-beta1-activated p38 mitogen-activated protein kinase in aortic smooth muscle cells. Arterioscler Thromb Vasc Biol.

[20] Jeong K, Murphy JM, Kim JH, Campbell PM, Park H, Rodriguez YAR (2021). FAK activation promotes SMC dedifferentiation via increased DNA methylation in contractile genes. Circ Res.

[21] Farina FM, Serio S, Hall IF, Zani S, Cassanmagnago GA, Climent M (2022). The epigenetic enzyme DOT1L orchestrates vascular smooth muscle cell-monocyte crosstalk and protects against atherosclerosis via the NF-kappaB pathway. Eur Heart J.

[22] Zhang X, Sun J, Canfrán-Duque A, Aryal B, Tellides G, Chang YJ (2021). Deficiency of histone lysine methyltransferase SETDB2 in hematopoietic cells promotes vascular inflammation and accelerates atherosclerosis. JCI Insight.

[23] Zhang X, He D, Xiang Y, Wang C, Liang B, Li B (2022). DYSF promotes monocyte activation in atherosclerotic cardiovascular disease as a DNA methylation-driven gene. Transl Res.

[24] Xie L, Ding N, Zhang H, Liu K, Xiong J, Ma S (2021). SNF5 promotes IL-1beta expression via H3K4me1 in atherosclerosis induced by homocysteine. Int J Biochem Cell Biol.

[25] Ma S, Lu G, Zhang Q, Ding N, Jie Y, Zhang H (2022). Extracellular-superoxide dismutase DNA methylation promotes oxidative stress in homocysteine-induced atherosclerosis. Acta Biochim Biophys Sin (Shanghai).

[26] Fang Y, Li J, Niu X, Ma N, Zhao J (2021). Hypomethylation of Rnase6 promoter enhances proliferation and migration of murine aortic vascular smooth muscle cells and aggravates atherosclerosis in mice. Front Bioeng Biotechnol.

[27] Huang G, Cheng Z, Hildebrand A, Wang C, Cimini M, Roy R (2022). Diabetes impairs cardioprotective function of endothelial progenitor cell-derived extracellular vesicles via H3K9Ac inhibition. Theranostics.

[28] Schiano C, Balbi C, Burrello J, Ruocco A, Infante T, Fiorito C (2022). De novo DNA methylation induced by circulating extracellular vesicles from acute coronary syndrome patients. Atherosclerosis.

[29] Lan C, Chen C, Qu S, Cao N, Luo H, Yu C (2022). Inhibition of DYRK1A, via histone modification, promotes cardiomyocyte cell cycle activation and cardiac repair after myocardial infarction. EBioMedicine.

[30] Ackeifi C, Wang P, Karakose E, Manning Fox JE, González BJ, Liu H (2020). GLP-1 receptor agonists synergize with DYRK1A inhibitors to potentiate functional human β cell regeneration. Sci Transl Med.

[31] Wang P, Alvarez-Perez JC, Felsenfeld DP, Liu H, Sivendran S, Bender A (2015). A high-throughput chemical screen reveals that harmine-mediated inhibition of DYRK1A increases human pancreatic beta cell replication. Nat Med.

[32] Watson CJ, Collier P, Tea I, Neary R, Watson JA, Robinson C (2014). Hypoxia-induced epigenetic modifications are associated with cardiac tissue fibrosis and the development of a myofibroblast-like phenotype. Hum Mol Genet.

[33] Rigaud VO, Zarka C, Kurian J, Harlamova D, Elia A, Kasatkin N (2022). UCP2 modulates cardiomyocyte cell cycle activity, acetyl-CoA, and histone acetylation in response to moderate hypoxia. JCI Insight.

[34] Lei I, Tian S, Gao W, Liu L, Guo Y, Tang P (2021). Acetyl-CoA production by specific metabolites promotes cardiac repair after myocardial infarction via histone acetylation. Elife.

[35] Zhang Z, Ding S, Wang Z, Zhu X, Zhou Z, Zhang W (2022). Prmt1 upregulated by Hdc deficiency aggravates acute myocardial infarction via NETosis. Acta Pharm Sin B.

[36] Li Y, Quan X, Li X, Pan Y, Zhang T, Liang Z (2019). Kdm6A protects against hypoxia-induced cardiomyocyte apoptosis via H3K27me3 demethylation of Ncx gene. J Cardiovasc Transl Res.

[37] Cao Y, Luo F, Peng J, Fang Z, Liu Q, Zhou S (2022). KMT2B-dependent RFK transcription activates the TNF-alpha/NOX2 pathway and enhances ferroptosis caused by myocardial ischemia-reperfusion. J Mol Cell Cardiol.

[38] Zhang B, Liu G, Huang B, Liu H, Jiang H, Hu Z (2022). KDM3A attenuates myocardial ischemic and reperfusion injury by ameliorating cardiac microvascular endothelial cell pyroptosis. Oxid Med Cell Longev.

[39] Wang S, Hu S, Luo X, Bao X, Li J, Liu M (2022). Prevalence and prognostic significance of DNMT3A- and TET2- clonal haematopoiesis-driver mutations in patients presenting with ST-segment elevation myocardial infarction. EBioMedicine.

[40] Li Y, Hiroi Y, Ngoy S, Okamoto R, Noma K, Wang CY (2011). Notch1 in bone marrow-derived cells mediates cardiac repair after myocardial infarction. Circulation.

[41] Li M, Jiao L, Shao Y, Li H, Sun L, Yu Q (2022). LncRNA-ZFAS1 promotes myocardial ischemia-reperfusion injury through DNA methylation-mediated Notch1 down-regulation in mice. JACC Basic Transl Sci.

[42] He Y, Ling S, Sun Y, Sheng Z, Chen Z, Pan X (2019). DNA methylation regulates alpha-smooth muscle actin expression during cardiac fibroblast differentiation. J Cell Physiol.

[43] Liang Q, Cai M, Zhang J, Song W, Zhu W, Xi L (2020). Role of muscle-specific histone methyltransferase (Smyd1) in exercise-induced cardioprotection against pathological remodeling after myocardial infarction. Int J Mol Sci.

[44] Rahm AK, Wieder T, Gramlich D, Muller ME, Wunsch MN, El Tahry FA (2021). Differential regulation of K(Ca) 2.1 (KCNN1) K(+) channel expression by histone deacetylases in atrial fibrillation with concomitant heart failure. Physiol Rep.

[45] Lamothe J, Khurana S, Tharmalingam S, Williamson C, Byrne CJ, Khaper N (2020). The role of DNMT and HDACs in the fetal programming of hypertension by glucocorticoids. Oxid Med Cell Longev.

[46] Lamothe J, Khurana S, Tharmalingam S, Williamson C, Byrne CJ, Lees SJ (2021). Oxidative stress mediates the fetal programming of hypertension by glucocorticoids. Antioxidants (Basel).

[47] Wang J, Cui J, Chen R, Deng Y, Liao X, Wei Y (2017). Prenatal exposure to lipopolysaccharide alters renal DNA methyltransferase expression in rat offspring. PLoS One.

[48] Liao Y, Chu C, Yan Y, Wang D, Ma Q, Gao K (2022). High dietary salt intake is associated with histone methylation in salt-sensitive individuals. Front Nutr.

[49] Wang N, Peng YJ, Su X, Prabhakar NR, Nanduri J (2021). Histone deacetylase 5 is an early epigenetic regulator of intermittent hypoxia induced sympathetic nerve activation and blood pressure. Front Physiol.

[50] Zhang X, Sun Y (2022). Chromodomain Helicase DNA Binding Protein 1-like, a negative regulator of Forkhead box O3a, promotes the proliferation and migration of Angiotensin II-induced vascular smooth muscle cells. Bioengineered.

[51] Chen H, Xu X, Liu Z, Wu Y (2021). MiR-22–3p suppresses vascular remodeling and oxidative stress by targeting CHD9 during the development of hypertension. J Vasc Res.

[52] Armando I, Cuevas S, Fan C, Kumar M, Izzi Z, Jose PA (2022). G protein-coupled receptor 37L1 modulates epigenetic changes in human renal proximal tubule cells. Int J Mol Sci.

[53] Gusterson RJ, Jazrawi E, Adcock IM, Latchman DS (2003). The transcriptional co-activators CREB-binding protein (CBP) and p300 play a critical role in cardiac hypertrophy that is dependent on their histone acetyltransferase activity. J Biol Chem.

[54] Duan Y, Zhou B, Su H, Liu Y, Du C (2013). miR-150 regulates high glucose-induced cardiomyocyte hypertrophy by targeting the transcriptional co-activator p300. Exp Cell Res.

[55] Zhou XL, Zhu RR, Wu X, Xu H, Li YY, Xu QR (2019). NSD2 promotes ventricular remodelling mediated by the regulation of H3K36me2. J Cell Mol Med.

[56] Wang YY, Gao B, Yang Y, Jia SB, Ma XP, Zhang MH (2022). Histone deacetylase 3 suppresses the expression of SHP-1 via deacetylation of DNMT1 to promote heart failure. Life Sci.

[57] Mehta G, Kumarasamy S, Wu J, Walsh A, Liu L, Williams K (2015). MITF interacts with the SWI/SNF subunit, BRG1, to promote GATA4 expression in cardiac hypertrophy. J Mol Cell Cardiol.

[58] Sunagawa Y, Katayama A, Funamoto M, Shimizu K, Shimizu S, Sari N (2022). The polyunsaturated fatty acids, EPA and DHA, ameliorate myocardial infarction-induced heart failure by inhibiting p300-HAT activity in rats. J Nutr Biochem.

[59] Sunagawa Y, Shimizu K, Katayama A, Funamoto M, Shimizu K, Nurmila S (2021). Metformin suppresses phenylephrine-induced hypertrophic responses by inhibiting p300-HAT activity in cardiomyocytes. J Pharmacol Sci.

[60] Suzuki H, Katanasaka Y, Sunagawa Y, Miyazaki Y, Funamoto M, Wada H (2016). Tyrosine phosphorylation of RACK1 triggers cardiomyocyte hypertrophy by regulating the interaction between p300 and GATA4. Biochim Biophys Acta.

[61] Qi L, Chi X, Zhang X, Feng X, Chu W, Zhang S (2019). Kindlin-2 suppresses transcription factor GATA4 through interaction with SUV39H1 to attenuate hypertrophy. Cell Death Dis.

[62] Yu L, Yang G, Weng X, Liang P, Li L, Li J (2015). Histone methyltransferase SET1 mediates angiotensin II-induced endothelin-1 transcription and cardiac hypertrophy in mice. Arterioscler Thromb Vasc Biol.

[63] Cai S, Wang P, Xie T, Li Z, Li J, Lan R (2020). Histone H4R3 symmetric di-methylation by Prmt5 protects against cardiac hypertrophy via regulation of Filip1L/beta-catenin. Pharmacol Res.

[64] He T, Huang J, Chen L, Han G, Stanmore D, Krebs-Haupenthal J (2020). Cyclic AMP represses pathological MEF2 activation by myocyte-specific hypo-phosphorylation of HDAC5. J Mol Cell Cardiol.

[65] Hu T, Schreiter FC, Bagchi RA, Tatman PD, Hannink M, McKinsey TA (2019). HDAC5 catalytic activity suppresses cardiomyocyte oxidative stress and NRF2 target gene expression. J Biol Chem.

[66] Yoon S, Kook T, Min HK, Kwon DH, Cho YK, Kim M (2018). PP2A negatively regulates the hypertrophic response by dephosphorylating HDAC2 S394 in the heart. Exp Mol Med.

[67] Gao W, Guo N, Zhao S, Chen Z, Zhang W, Yan F (2020). FBXW7 promotes pathological cardiac hypertrophy by targeting EZH2-SIX1 signaling. Exp Cell Res.

[68] Delgado-Olguin P, Huang Y, Li X, Christodoulou D, Seidman CE, Seidman JG (2012). Epigenetic repression of cardiac progenitor gene expression by Ezh2 is required for postnatal cardiac homeostasis. Nat Genet.

[69] Mathiyalagan P, Okabe J, Chang L, Su Y, Du XJ, El-Osta A (2014). The primary microRNA-208b interacts with Polycomb-group protein, Ezh2, to regulate gene expression in the heart. Nucleic Acids Res.

[70] Wang Z, Zhang XJ, Ji YX, Zhang P, Deng KQ, Gong J (2016). The long noncoding RNA Chaer defines an epigenetic checkpoint in cardiac hypertrophy. Nat Med.

[71] Madsen A, Krause J, Hoppner G, Hirt MN, Tan WLW, Lim I (2021). Hypertrophic signaling compensates for contractile and metabolic consequences of DNA methyltransferase 3A loss in human cardiomyocytes. J Mol Cell Cardiol.

[72] Chen K, Jian D, Zhao L, Zang X, Song W, Ma J (2019). Protective effect of histone methyltransferase NSD3 on ISO-induced cardiac hypertrophy. FEBS Lett.

[73] Zhang QJ, Chen HZ, Wang L, Liu DP, Hill JA, Liu ZP (2011). The histone trimethyllysine demethylase JMJD2A promotes cardiac hypertrophy in response to hypertrophic stimuli in mice. J Clin Invest.

[74] Zhang QJ, Tran TAT, Wang M, Ranek MJ, Kokkonen-Simon KM, Gao J (2018). Histone lysine dimethyl-demethylase KDM3A controls pathological cardiac hypertrophy and fibrosis. Nat Commun.

[75] Guo Z, Lu J, Li J, Wang P, Li Z, Zhong Y (2018). JMJD3 inhibition protects against isoproterenol-induced cardiac hypertrophy by suppressing beta-MHC expression. Mol Cell Endocrinol.

[76] Rosa-Garrido M, Chapski DJ, Schmitt AD, Kimball TH, Karbassi E, Monte E (2017). High-resolution mapping of chromatin conformation in cardiac myocytes reveals structural remodeling of the epigenome in heart failure. Circulation.

[77] Sundaresan NR, Vasudevan P, Zhong L, Kim G, Samant S, Parekh V (2012). The sirtuin SIRT6 blocks IGF-Akt signaling and development of cardiac hypertrophy by targeting c-Jun. Nat Med.

[78] Liao PP, Liu LH, Wang B, Fang X, Zhou SQ, Li W (2018). Correlation between histone deacetylase 9 and regulatory T cell in patients with chronic heart failure. Curr Med Sci.

[79] Hohl M, Wagner M, Reil JC, Muller SA, Tauchnitz M, Zimmer AM (2013). HDAC4 controls histone methylation in response to elevated cardiac load. J Clin Invest.

[80] Willis MS, Holley DW, Wang Z, Chen X, Quintana M, Jensen BC (2017). BRG1 and BRM function antagonistically with c-MYC in adult cardiomyocytes to regulate conduction and contractility. J Mol Cell Cardiol.

[81] Wang B, Tan Y, Zhang Y, Zhang S, Duan X, Jiang Y (2022). Loss of KDM5B ameliorates pathological cardiac fibrosis and dysfunction by epigenetically enhancing ATF3 expression. Exp Mol Med.

[82] Assmus B, Cremer S, Kirschbaum K, Culmann D, Kiefer K, Dorsheimer L (2021). Clonal haematopoiesis in chronic ischaemic heart failure: prognostic role of clone size for DNMT3A- and TET2-driver gene mutations. Eur Heart J.

[83] Abplanalp WT, Cremer S, John D, Hoffmann J, Schuhmacher B, Merten M (2021). Clonal hematopoiesis-driver DNMT3A mutations alter immune cells in heart failure. Circ Res.

[84] Syren P, Rahm AK, Schweizer PA, Bruehl C, Katus HA, Frey N (2021). Histone deacetylase 2-dependent ventricular electrical remodeling in a porcine model of early heart failure. Life Sci.

[85] Shen JF, Fan ZB, Wu CW, Qi GX, Cao QY, Xu F (2022). Sacubitril valsartan enhances cardiac function and alleviates myocardial infarction in rats through a SUV39H1/SPP1 axis. Oxid Med Cell Longev.

[86] Yang G, Zhang X, Weng X, Liang P, Dai X, Zeng S (2017). SUV39H1 mediated SIRT1 trans-repression contributes to cardiac ischemia-reperfusion injury. Basic Res Cardiol.

[87] Song T, Guan X, Wang X, Qu S, Zhang S, Hui W (2021). Dynamic modulation of gut microbiota improves post-myocardial infarct tissue repair in rats via butyric acid-mediated histone deacetylase inhibition. FASEB J.

[88] Liu Z, Zhang Y, Qiu C, Zhu H, Pan S, Jia H (2020). Diabetes mellitus exacerbates post-myocardial infarction heart failure by reducing sarcolipin promoter methylation. ESC Heart Fail.

[89] Glaser K, Schepers EJ, Zwolshen HM, Lake CM, Timchenko NA, Karns RA (2024). EZH2 is a key component of hepatoblastoma tumor cell growth. Pediatr Blood Cancer.

[90] Kent D, Marchetti L, Mikulasova A, Russell LJ, Rico D (2023). Broad H3K4me3 domains: maintaining cellular identity and their implication in super-enhancer hijacking. Bioessays.

[91] Li W, Zhou C, Yu L, Hou Z, Liu H, Kong L (2024). Tumor-derived lactate promotes resistance to bevacizumab treatment by facilitating autophagy enhancer protein RUBCNL expression through histone H3 lysine 18 lactylation (H3K18la) in colorectal cancer. Autophagy.

[92] Li QL, Wang DY, Ju LG, Yao J, Gao C, Lei PJ (2019). The hyper-activation of transcriptional enhancers in breast cancer. Clin Epigenetics.

[93] Jing N, Zhang K, Chen X, Liu K, Wang J, Xiao L (2023). ADORA2A-driven proline synthesis triggers epigenetic reprogramming in neuroendocrine prostate and lung cancers. J Clin Invest.

[94] Durall RT, Huang J, Wojenski L, Huang Y, Gokhale PC, Leeper BA (2023). The BRD4-NUT fusion alone drives malignant transformation of NUT carcinoma. Cancer Res.

[95] Bai XY, Li S, Wang M, Li X, Yang Y, Xu Z (2018). Krüppel-like factor 9 down-regulates matrix metalloproteinase 9 transcription and suppresses human breast cancer invasion. Cancer Lett.

[96] Tung B, Ma D, Wang S, Oyinlade O, Laterra J, Ying M (2018). Krüppel-like factor 9 and histone deacetylase inhibitors synergistically induce cell death in glioblastoma stem-like cells. BMC Cancer.

[97] Sekine S, Tagami S, Yokoyama S (2012). Structural basis of transcription by bacterial and eukaryotic RNA polymerases. Curr Opin Struct Biol.

[98] Wei T, Lin R, Fu X, Lu Y, Zhang W, Li Z (2022). Epigenetic regulation of the DNMT1/MT1G/KLF4/CA9 axis synergises the anticancer effects of sorafenib in hepatocellular carcinoma. Pharmacol Res.

[99] Yu T, Chen X, Zhang W, Liu J, Avdiushko R, Napier DL (2016). KLF4 regulates adult lung tumor-initiating cells and represses K-Ras-mediated lung cancer. Cell Death Differ.

[100] Perez MF, Sarkies P (2023). Histone methyltransferase activity affects metabolism in human cells independently of transcriptional regulation. PLoS Biol.

[101] Parra A, Rabin R, Pappas J, Pascual P, Cazalla M, Arias P (2023). Clinical heterogeneity and different phenotypes in patients with SETD2 variants: 18 new patients and review of the literature. Genes (Basel).

[102] Romero VI, Arias-Almeida B, Aguiar SA (2022). NSD1 gene evolves under episodic selection within primates and mutations of specific exons in humans cause Sotos syndrome. BMC Genomics.

[103] Chen TJ, Hung HS, Cheng TL, Wang DC (2024). Histone deacetylase inhibitor attenuates the effects of 27-hydroxycholesterol on the rat brain. Neurosci Lett.

[104] Marinho D, Ferreira IL, Lorenzoni R, Cardoso SM, Santana I, Rego AC (2023). Reduction of class I histone deacetylases ameliorates ER-mitochondria cross-talk in Alzheimer’s disease. Aging Cell.

[105] Wang C, Shen D, Hu Y, Chen J, Liu J, Huang Y (2023). Selective targeting of class I HDAC reduces microglial inflammation in the entorhinal cortex of young APP/PS1 mice. Int J Mol Sci.

[106] Dai Y, Wei T, Huang Y, Bei Y, Lin H, Shen Z (2023). Upregulation of HDAC9 in hippocampal neurons mediates depression-like behaviours by inhibiting ANXA2 degradation. Cell Mol Life Sci.

[107] Baek SY, Lee J, Kim T, Lee H, Choi HS, Park H (2023). Development of a novel histone deacetylase inhibitor unveils the role of HDAC11 in alleviating depression by inhibition of microglial activation. Biomed Pharmacother.

[108] Shang W, Zhao X, Yang F, Wang D, Lu L, Xu Z (2022). Ginsenoside Rg1 nanoparticles induce demethylation of H3K27me3 in VEGF-A and Jagged 1 promoter regions to activate angiogenesis after ischemic stroke. Int J Nanomedicine.

[109] Martin LJ, Adams DA, Niedzwiecki MV, Wong M (2022). Aberrant DNA and RNA methylation occur in spinal cord and skeletal muscle of human SOD1 mouse models of ALS and in human ALS: targeting DNA methylation is therapeutic. Cells.

[110] Raghu D, Xue HH, Mielke LA (2019). Control of lymphocyte fate, infection, and tumor immunity by TCF-1. Trends Immunol.

[111] Li F, Zhao X, Zhang Y, Shao P, Ma X, Paradee WJ (2021). T(FH) cells depend on Tcf1-intrinsic HDAC activity to suppress CTLA4 and guard B-cell help function. Proc Natl Acad Sci U S A.

[112] Gullicksrud JA, Li F, Xing S, Zeng Z, Peng W, Badovinac VP (2017). Differential requirements for Tcf1 long isoforms in CD8(+) and CD4(+) T cell responses to acute viral infection. J Immunol.

[113] Bélanger S, Haupt S, Faliti CE, Getzler A, Choi J, Diao H (2023). The chromatin regulator Mll1 supports T follicular helper cell differentiation by controlling expression of Bcl6, LEF-1, and TCF-1. J Immunol.

[114] Thio CL, Chi PY, Lai AC, Chang YJ (2018). Regulation of type 2 innate lymphoid cell-dependent airway hyperreactivity by butyrate. J Allergy Clin Immunol.

[115] Ghiboub M, Zhao J, Li Yim AYF, Schilderink R, Verseijden C, van Hamersveld PHP (2020). HDAC3 mediates the inflammatory response and LPS tolerance in human monocytes and macrophages. Front Immunol.

[116] Wisler JR, Singh K, McCarty A, Harkless R, Karpurapu M, Hernandez E (2022). Exosomal transfer of DNA methyl-transferase mRNA induces an immunosuppressive phenotype in human monocytes. Shock.

[117] Chen J, Chen ZJ (2018). PtdIns4P on dispersed trans-Golgi network mediates NLRP3 inflammasome activation. Nature.

[118] Zhong W, Li B, Xu Y, Yang P, Chen R, Wang Z (2018). Hypermethylation of the micro-RNA 145 promoter is the key regulator for NLRP3 inflammasome-induced activation and plaque formation. JACC Basic Transl Sci.

[119] Bockstiegel J, Wurnig SL, Engelhardt J, Enns J, Hansen FK, Weindl G (2023). Pharmacological inhibition of HDAC6 suppresses NLRP3 inflammasome-mediated IL-1β release. Biochem Pharmacol.

[120] Moreira JD, Iakhiaev A, Vankayalapati R, Jung BG, Samten B (2022). Histone deacetylase-2 controls IL-1β production through the regulation of NLRP3 expression and activation in tuberculosis infection. iScience.

[121] Tang SC, Yeh JI, Hung SJ, Hsiao YP, Liu FT, Yang JH (2016). Glycolic acid silences inflammasome complex genes, NLRC4 and ASC, by inducing DNA methylation in HaCaT cells. DNA Cell Biol.

[122] Gillette TG, Hill JA (2015). Readers, writers, and erasers: chromatin as the whiteboard of heart disease. Circ Res.

[123] Marmorstein R, Zhou MM (2014). Writers and readers of histone acetylation: structure, mechanism, and inhibition. Cold Spring Harb Perspect Biol.

[124] Capone V, Della Torre L, Carannante D, Babaei M, Altucci L, Benedetti R (2023). HAT1: landscape of biological function and role in cancer. Cells.

[125] Militi S, Nibhani R, Jalali M, Pauklin S (2023). RBL2-E2F-GCN5 guide cell fate decisions during tissue specification by regulating cell-cycle-dependent fluctuations of non-cell-autonomous signaling. Cell Rep.

[126] Zhou WH, Luo Y, Li RX, Degrace P, Jourdan T, Qiao F (2023). Inhibition of mitochondrial fatty acid β-oxidation activates mTORC1 pathway and protein synthesis via Gcn5-dependent acetylation of Raptor in zebrafish. J Biol Chem.

[127] Xie M, Hill JA (2013). HDAC-dependent ventricular remodeling. Trends Cardiovasc Med.

[128] Lu Y, Yang S (2009). Angiotensin II induces cardiomyocyte hypertrophy probably through histone deacetylases. Tohoku J Exp Med.

[129] Liu Y, Wang DL, Chen S, Zhao L, Sun FL (2012). Oncogene Ras/phosphatidylinositol 3-kinase signaling targets histone H3 acetylation at lysine 56. J Biol Chem.

[130] Latcheva NK, Delaney TL, Viveiros JM, Smith RA, Bernard KM, Harsin B (2019). The CHD protein, kismet, is important for the recycling of synaptic vesicles during endocytosis. Sci Rep.

[131] Havas AP, Tula-Sanchez AA, Steenhoek HM, Bhakta A, Wingfield T, Huntley MJ (2024). Defining cellular responses to HDAC-selective inhibitors reveals that efficient targeting of HDAC3 is required to elicit cytotoxicity and overcome naïve resistance to pan-HDACi in diffuse large B cell lymphoma. Transl Oncol.

[132] Farhadipour M, Arnauts K, Clarysse M, Thijs T, Liszt K, Van der Schueren B (2023). SCFAs switch stem cell fate through HDAC inhibition to improve barrier integrity in 3D intestinal organoids from patients with obesity. iScience.

[133] Deng J, Hou B, Hou X, Chen Y, Zhang T, Chen H (2023). Discovery of benzamide-based PI3K/HDAC dual inhibitors with marked pro-apoptosis activity in lymphoma cells. Eur J Med Chem.

[134] Roeschert I, Poon E, Henssen AG, Garcia HD, Gatti M, Giansanti C (2021). Combined inhibition of Aurora-A and ATR kinase results in regression of MYCN-amplified neuroblastoma. Nat Cancer.

[135] Li C, Liao J, Wang X, Chen FX, Guo X, Chen X (2023). Combined Aurora kinase A and CHK1 inhibition enhances radiosensitivity of triple-negative breast cancer through induction of apoptosis and mitotic catastrophe associated with excessive DNA damage. Int J Radiat Oncol Biol Phys.

[136] Varshney N, Murmu S, Baral B, Kashyap D, Singh S, Kandpal M (2023). Unraveling the Aurora kinase A and Epstein-Barr nuclear antigen 1 axis in Epstein Barr virus associated gastric cancer. Virology.

[137] Hickenlooper SM, Davis K, Szulik MW, Sheikh H, Miller M, Valdez S (2022). Histone H4K20 trimethylation is decreased in murine models of heart disease. ACS Omega.

[138] Verma A, Arya R, Brahmachari V (2022). Identification of a polycomb responsive region in human HoxA cluster and its long-range interaction with polycomb enriched genomic regions. Gene.

[139] Husmann D, Gozani O (2019). Histone lysine methyltransferases in biology and disease. Nat Struct Mol Biol.

[140] Ning J, Chen L, Xiao G, Zeng Y, Shi W, Tanzhu G (2023). The protein arginine methyltransferase family (PRMTs) regulates metastases in various tumors: from experimental study to clinical application. Biomed Pharmacother.

[141] Cao N, Lan C, Chen C, Xu Z, Luo H, Zheng S (2022). Prenatal lipopolysaccharides exposure induces transgenerational inheritance of hypertension. Circulation.

[142] Zhang SM, Cai WL, Liu X, Thakral D, Luo J, Chan LH (2021). KDM5B promotes immune evasion by recruiting SETDB1 to silence retroelements. Nature.

[143] Sun Y, Jin X, Meng J, Guo F, Chen T, Zhao X (2023). MST2 methylation by PRMT5 inhibits Hippo signaling and promotes pancreatic cancer progression. EMBO J.

[144] Izumi T, Rychahou P, Chen L, Smith MH, Valentino J (2023). Copy number variation that influences the ionizing radiation sensitivity of oral squamous cell carcinoma. Cells.

[145] Sloan SL, Brown F, Long M, Weigel C, Koirala S, Chung JH (2023). PRMT5 supports multiple oncogenic pathways in mantle cell lymphoma. Blood.

[146] Kim KH, Oprescu SN, Snyder MM, Kim A, Jia Z, Yue F (2023). PRMT5 mediates FoxO1 methylation and subcellular localization to regulate lipophagy in myogenic progenitors. Cell Rep.

[147] Ye S, Tian X, Anwaier A, Wei S, Liu W, Su J (2023). Protein arginine methyltransferases refine the classification of clear cell renal cell carcinoma with distinct prognosis and tumor microenvironment characteristics. Int J Biol Sci.

[148] Shi Y, Zhang H, Huang S, Yin L, Wang F, Luo P (2022). Epigenetic regulation in cardiovascular disease: mechanisms and advances in clinical trials. Signal Transduct Target Ther.

[149] Moore-Morris T, van Vliet PP, Andelfinger G, Puceat M (2018). Role of epigenetics in cardiac development and congenital diseases. Physiol Rev.

[150] Li B, Liang Y, Bao H, Li D, Zhang Y, Dun X (2023). Real-ambient particulate matter exposure-induced FGFR1 methylation contributes to cardiac dysfunction via lipid metabolism disruption. Sci Total Environ.

[151] Wang X, Li Q, He S, Bai J, Ma C, Zhang L (2022). LncRNA FENDRR with m6A RNA methylation regulates hypoxia-induced pulmonary artery endothelial cell pyroptosis by mediating DRP1 DNA methylation. Mol Med.

[152] Shen J, Jiao Y, Ding N, Xie L, Ma S, Zhang H (2022). Homocysteine facilitates endoplasmic reticulum stress and apoptosis of hepatocytes by suppressing ERO1α expression via cooperation between DNMT1 and G9a. Cell Biol Int.

[153] Xie L, Ma S, Ding N, Wang Y, Lu G, Xu L (2021). Homocysteine induces podocyte apoptosis by regulating miR-1929-5p expression through c-Myc, DNMT1 and EZH2. Mol Oncol.

[154] Vieira JM, Howard S, Villa Del Campo C, Bollini S, Dube KN, Masters M (2017). BRG1-SWI/SNF-dependent regulation of the Wt1 transcriptional landscape mediates epicardial activity during heart development and disease. Nat Commun.

[155] Messina G, Prozzillo Y, Delle Monache F, Santopietro MV, Atterrato MT, Dimitri P (2021). The ATPase SRCAP is associated with the mitotic apparatus, uncovering novel molecular aspects of Floating-Harbor syndrome. BMC Biol.

[156] Yan S, Thienthanasit R, Chen D, Engelen E, Bruhl J, Crossman DK (2020). CHD7 regulates cardiovascular development through ATP-dependent and -independent activities. Proc Natl Acad Sci U S A.

[157] Zhou B, Wang L, Zhang S, Bennett BD, He F, Zhang Y (2016). INO80 governs superenhancer-mediated oncogenic transcription and tumor growth in melanoma. Genes Dev.

[158] Sokolova V, Lee G, Mullins A, Mody P, Watanabe S, Tan D (2023). DNA-translocation-independent role of INO80 remodeler in DNA damage repairs. J Biol Chem.

[159] Davó-Martínez C, Helfricht A, Ribeiro-Silva C, Raams A, Tresini M, Uruci S (2023). Different SWI/SNF complexes coordinately promote R-loop- and RAD52-dependent transcription-coupled homologous recombination. Nucleic Acids Res.

[160] Higuchi S, Suehiro Y, Izuhara L, Yoshina S, Hirasawa A, Mitani S (2023). BCL7B, a SWI/SNF complex subunit, orchestrates cancer immunity and stemness. BMC Cancer.

[161] Li X, Wang S, Yu X, Li S (2023). Transcriptional regulation of autophagy by chromatin remodeling complex and histone variant. Autophagy.

[162] Guo C, Zhang Y, Tan R, Tang Z, Lam CM, Ye X (2022). Arid1a regulates bladder urothelium formation and maintenance. Dev Biol.

[163] Zhou Z, Zhou Y, Liu W, Dai J (2023). A novel cuproptosis-related lncRNAs signature predicts prognostic and immune of bladder urothelial carcinoma. Front Genet.

[164] Rehman H, Chandrashekar DS, Balabhadrapatruni C, Nepal S, Balasubramanya SAH, Shelton AK (2022). ARID1A-deficient bladder cancer is dependent on PI3K signaling and sensitive to EZH2 and PI3K inhibitors. JCI Insight.

[165] Huang R, Wu D, Zhang K, Hu G, Liu Y, Jiang Y (2023). ARID1A loss induces P4HB to activate fibroblasts to support lung cancer cell growth, invasion, and chemoresistance. Cancer Sci.

[166] Sun D, Qian H, Wang J, Xie T, Teng F, Li J (2022). ARID1A deficiency reverses the response to anti-PD(L)1 therapy in EGFR-mutant lung adenocarcinoma by enhancing autophagy-inhibited type I interferon production. Cell Commun Signal.

[167] Creyghton MP, Cheng AW, Welstead GG, Kooistra T, Carey BW, Steine EJ (2010). Histone H3K27ac separates active from poised enhancers and predicts developmental state. Proc Natl Acad Sci U S A.

[168] Boileau RM, Chen KX, Blelloch R (2023). Loss of MLL3/4 decouples enhancer H3K4 monomethylation, H3K27 acetylation, and gene activation during embryonic stem cell differentiation. Genome Biol.

[169] Kreibich E, Kleinendorst R, Barzaghi G, Kaspar S, Krebs AR (2023). Single-molecule footprinting identifies context-dependent regulation of enhancers by DNA methylation. Mol Cell.

[170] Whalen CK, Henker B, Granger DA (1990). Social judgment processes in hyperactive boys: effects of methylphenidate and comparisons with normal peers. J Abnorm Child Psychol.

[171] Tam PLF, Cheung MF, Chan LY, Leung D (2024). Cell-type differential targeting of SETDB1 prevents aberrant CTCF binding, chromatin looping, and cis-regulatory interactions. Nat Commun.

[172] Tang R, Li Y, Han F, Li Z, Lin X, Sun H (2021). A CTCF-binding element and histone deacetylation cooperatively maintain chromatin loops, linking to long-range gene regulation in cancer genomes. Front Oncol.

[173] Min HY, Lee SC, Woo JK, Jung HJ, Park KH, Jeong HM (2017). Essential role of DNA methyltransferase 1-mediated transcription of insulin-like growth factor 2 in resistance to histone deacetylase inhibitors. Clin Cancer Res.

[174] Valletta M, Russo R, Baglivo I, Russo V, Ragucci S, Sandomenico A (2020). Exploring the interaction between the SWI/SNF chromatin remodeling complex and the zinc finger factor CTCF. Int J Mol Sci.

[175] Ge Z, Yin C, Li Y, Tian D, Xiang Y, Li Q (2022). Long noncoding RNA NEAT1 promotes cardiac fibrosis in heart failure through increased recruitment of EZH2 to the Smad7 promoter region. J Transl Med.

[176] Lin JM, Hsu CH, Chen JC, Kao SH, Lin YC (2021). BCL-6 promotes the methylation of miR-34a by recruiting EZH2 and upregulating CTRP9 to protect ischemic myocardial injury. Biofactors.

[177] Zhang C, Ge S, Gong W, Xu J, Guo Z, Liu Z (2020). LncRNA ANRIL acts as a modular scaffold of WDR5 and HDAC3 complexes and promotes alteration of the vascular smooth muscle cell phenotype. Cell Death Dis.

[178] Stoll S, Wang C, Qiu H (2018). DNA methylation and histone modification in hypertension. Int J Mol Sci.

[179] Liaw A, Liu C, Ivanovski S, Han P (2022). The relevance of DNA methylation and histone modification in periodontitis: a scoping review. Cells.

[180] Xu S, Pelisek J, Jin ZG (2018). Atherosclerosis is an epigenetic disease. Trends Endocrinol Metab.

[181] Van Loo KMJ, Carvill GL, Becker AJ, Conboy K, Goldman AM, Kobow K (2022). Epigenetic genes and epilepsy - emerging mechanisms and clinical applications. Nat Rev Neurol.

[182] Esteller M (2007). Cancer epigenomics: DNA methylomes and histone-modification maps. Nat Rev Genet.

[183] Phasaludeen B, Emerald BS, Ansari SA (2022). The epigenetic-metabolic interplay in gliomagenesis. Open Biol.

[184] Thomson S, Clayton AL, Mahadevan LC (2001). Independent dynamic regulation of histone phosphorylation and acetylation during immediate-early gene induction. Mol Cell.

[185] Cheung P, Tanner KG, Cheung WL, Sassone-Corsi P, Denu JM, Allis CD (2000). Synergistic coupling of histone H3 phosphorylation and acetylation in response to epidermal growth factor stimulation. Mol Cell.

[186] Manning LR, Manning JM (2018). Contributions to nucleosome dynamics in chromatin from interactive propagation of phosphorylation/acetylation and inducible histone lysine basicities. Protein Sci.

[187] Fischle W, Wang Y, Allis CD (2003). Binary switches and modification cassettes in histone biology and beyond. Nature.

[188] Terada Y (2006). Aurora-B/AIM-1 regulates the dynamic behavior of HP1alpha at the G2-M transition. Mol Biol Cell.

[189] Demidov D, Hesse S, Tewes A, Rutten T, Fuchs J, Ashtiyani RK (2009). Aurora1 phosphorylation activity on histone H3 and its cross-talk with other post-translational histone modifications in Arabidopsis. Plant J.

[190] Nepali K, Liou JP (2021). Recent developments in epigenetic cancer therapeutics: clinical advancement and emerging trends. J Biomed Sci.

[191] Eaton DM, Martin TG, Kasa M, Djalinac N, Ljubojevic-Holzer S, Von Lewinski D (2022). HDAC inhibition regulates cardiac function by increasing myofilament calcium sensitivity and decreasing diastolic tension. Pharmaceutics.

[192] Wallner M, Eaton DM, Berretta RM, Liesinger L, Schittmayer M, Gindlhuber J (2020). HDAC inhibition improves cardiopulmonary function in a feline model of diastolic dysfunction. Sci Transl Med.

[193] Benza RL, Adamson PB, Bhatt DL, Frick F, Olsson G, Bergh N (2024). CS1, a controlled-release formulation of valproic acid, for the treatment of patients with pulmonary arterial hypertension: rationale and design of a Phase 2 clinical trial. Pulm Circ.

[194] Liu F, Liu C, Chai Q, Zhao C, Meng H, Xue X (2023). Discovery of the first irreversible HDAC6 isoform selective inhibitor with potent anti-multiple myeloma activity. J Med Chem.

[195] Kumbhar N, Nimal S, Patil D, Kaiser VF, Haupt J, Gacche RN (2023). Repurposing of neprilysin inhibitor ‘sacubitrilat’ as an anti-cancer drug by modulating epigenetic and apoptotic regulators. Sci Rep.

[196] Yu T, Wang C, Yang J, Guo Y, Wu Y, Li X (2017). Metformin inhibits SUV39H1-mediated migration of prostate cancer cells. Oncogenesis.

[197] Tang G, Guo J, Zhu Y, Huang Z, Liu T, Cai J (2018). Metformin inhibits ovarian cancer via decreasing H3K27 trimethylation. Int J Oncol.

[198] Davies G, Lobanova L, Dawicki W, Groot G, Gordon JR, Bowen M (2017). Metformin inhibits the development, and promotes the resensitization, of treatment-resistant breast cancer. PLoS One.

[199] Zhang K, Huang R, Ji M, Lin S, Lai F, Wu D (2024). Rational design and optimization of novel 4-methyl quinazoline derivatives as PI3K/HDAC dual inhibitors with benzamide as zinc binding moiety for the treatment of acute myeloid leukemia. Eur J Med Chem.

[200] Katagiri T, Sunagawa Y, Maekawa T, Funamoto M, Shimizu S, Shimizu K (2022). Ecklonia stolonifera Okamura extract suppresses myocardial infarction-induced left ventricular systolic dysfunction by inhibiting p300-HAT activity. Nutrients.

[201] Sunagawa Y, Funamoto M, Shimizu K, Shimizu S, Sari N, Katanasaka Y (2021). Curcumin, an inhibitor of p300-HAT activity, suppresses the development of hypertension-induced left ventricular hypertrophy with preserved ejection fraction in Dahl rats. Nutrients.

[202] Sunagawa Y, Morimoto T, Wada H, Takaya T, Katanasaka Y, Kawamura T (2011). A natural p300-specific histone acetyltransferase inhibitor, curcumin, in addition to angiotensin-converting enzyme inhibitor, exerts beneficial effects on left ventricular systolic function after myocardial infarction in rats. Circ J.

[203] Evans LW, Bender A, Burnett L, Godoy L, Shen Y, Staten D (2020). Emodin and emodin-rich rhubarb inhibits histone deacetylase (HDAC) activity and cardiac myocyte hypertrophy. J Nutr Biochem.

[204] Rai R, Verma SK, Kim D, Ramirez V, Lux E, Li C (2017). A novel acetyltransferase p300 inhibitor ameliorates hypertension-associated cardio-renal fibrosis. Epigenetics.

[205] Rai R, Sun T, Ramirez V, Lux E, Eren M, Vaughan DE (2019). Acetyltransferase p300 inhibitor reverses hypertension-induced cardiac fibrosis. J Cell Mol Med.

[206] Jin L, Piao ZH, Liu CP, Sun S, Liu B, Kim GR (2018). Gallic acid attenuates calcium calmodulin-dependent kinase II-induced apoptosis in spontaneously hypertensive rats. J Cell Mol Med.

[207] Selvakumar P, Badgeley A, Murphy P, Anwar H, Sharma U, Lawrence K (2020). Flavonoids and other polyphenols act as epigenetic modifiers in breast cancer. Nutrients.

[208] Fernandes GFS, Silva GDB, Pavan AR, Chiba DE, Chin CM, Dos Santos JL (2017). Epigenetic regulatory mechanisms induced by resveratrol. Nutrients.

[209] Tan CS, Ho CF, Heng SS, Wu JS, Tan BK, Ng YK (2016). Clinacanthus nutans extracts modulate epigenetic link to cytosolic phospholipase A2 expression in SH-SY5Y cells and primary cortical neurons. Neuromolecular Med.

[210] Peng Y, Wang Y, Tang N, Sun D, Lan Y, Yu Z (2018). Andrographolide inhibits breast cancer through suppressing COX-2 expression and angiogenesis via inactivation of p300 signaling and VEGF pathway. J Exp Clin Cancer Res.

[211] Zhang H, Cai J, Li C, Deng L, Zhu H, Huang T (2023). Wogonin inhibits latent HIV-1 reactivation by downregulating histone crotonylation. Phytomedicine.

[212] Nardi I, Reno T, Yun X, Sztain T, Wang J, Dai H (2018). Triptolide inhibits Wnt signaling in NSCLC through upregulation of multiple Wnt inhibitory factors via epigenetic modifications to Histone H3. Int J Cancer.

[213] Furlan A, Monzani V, Reznikov LL, Leoni F, Fossati G, Modena D (2011). Pharmacokinetics, safety and inducible cytokine responses during a phase 1 trial of the oral histone deacetylase inhibitor ITF2357 (givinostat). Mol Med.

[214] Rambaldi A, Iurlo A, Vannucchi AM, Martino B, Guarini A, Ruggeri M (2021). Long-term safety and efficacy of givinostat in polycythemia vera: 4-year mean follow up of three phase 1/2 studies and a compassionate use program. Blood Cancer J.

[215] Huang HM, Fan SJ, Zhou XR, Liu YJ, Li X, Liao LP (2022). Histone deacetylase inhibitor givinostat attenuates nonalcoholic steatohepatitis and liver fibrosis. Acta Pharmacol Sin.

[216] Joosten LA, Leoni F, Meghji S, Mascagni P (2011). Inhibition of HDAC activity by ITF2357 ameliorates joint inflammation and prevents cartilage and bone destruction in experimental arthritis. Mol Med.

[217] Christensen DP, Gysemans C, Lundh M, Dahllöf MS, Noesgaard D, Schmidt SF (2014). Lysine deacetylase inhibition prevents diabetes by chromatin-independent immunoregulation and β-cell protection. Proc Natl Acad Sci U S A.

[218] Shein NA, Grigoriadis N, Alexandrovich AG, Simeonidou C, Lourbopoulos A, Polyzoidou E (2009). Histone deacetylase inhibitor ITF2357 is neuroprotective, improves functional recovery, and induces glial apoptosis following experimental traumatic brain injury. FASEB J.

[219] Zichittella C, Loria M, Celesia A, Di Liberto D, Corrado C, Alessandro R (2023). Long non-coding RNA H19 enhances the pro-apoptotic activity of ITF2357 (a histone deacetylase inhibitor) in colorectal cancer cells. Front Pharmacol.

[220] Todoerti K, Barbui V, Pedrini O, Lionetti M, Fossati G, Mascagni P (2010). Pleiotropic anti-myeloma activity of ITF2357: inhibition of interleukin-6 receptor signaling and repression of miR-19a and miR-19b. Haematologica.

[221] Savino AM, Sarno J, Trentin L, Vieri M, Fazio G, Bardini M (2017). The histone deacetylase inhibitor givinostat (ITF2357) exhibits potent anti-tumor activity against CRLF2-rearranged BCP-ALL. Leukemia.

[222] Armeanu S, Pathil A, Venturelli S, Mascagni P, Weiss TS, Göttlicher M (2005). Apoptosis on hepatoma cells but not on primary hepatocytes by histone deacetylase inhibitors valproate and ITF2357. J Hepatol.

[223] Milan M, Pace V, Maiullari F, Chirivì M, Baci D, Maiullari S (2018). Givinostat reduces adverse cardiac remodeling through regulating fibroblasts activation. Cell Death Dis.

[224] Jeong MY, Lin YH, Wennersten SA, Demos-Davies KM, Cavasin MA, Mahaffey JH (2018). Histone deacetylase activity governs diastolic dysfunction through a nongenomic mechanism. Sci Transl Med.

[225] Travers JG, Wennersten SA, Peña B, Bagchi RA, Smith HE, Hirsch RA (2021). HDAC inhibition reverses preexisting diastolic dysfunction and blocks covert extracellular matrix remodeling. Circulation.

[226] Badamchi-Zadeh A, Moynihan KD, Larocca RA, Aid M, Provine NM, Iampietro MJ (2018). Combined HDAC and BET inhibition enhances melanoma vaccine immunogenicity and efficacy. J Immunol.

[227] Odunsi K, Matsuzaki J, James SR, Mhawech-Fauceglia P, Tsuji T, Miller A (2014). Epigenetic potentiation of NY-ESO-1 vaccine therapy in human ovarian cancer. Cancer Immunol Res.

[228] Gatti-Mays ME, Gameiro SR, Ozawa Y, Knudson KM, Hicks KC, Palena C (2020). Improving the odds in advanced breast cancer with combination immunotherapy: stepwise addition of vaccine, immune checkpoint inhibitor, chemotherapy, and HDAC inhibitor in advanced stage breast cancer. Front Oncol.

[229] Scholz B, Schulte JS, Hamer S, Himmler K, Pluteanu F, Seidl MD (2019). HDAC (histone deacetylase) inhibitor valproic acid attenuates atrial remodeling and delays the onset of atrial fibrillation in mice. Circ Arrhythm Electrophysiol.

[230] Wang P, Fan F, Li X, Sun X, Ma L, Wu J (2018). Riboflavin attenuates myocardial injury via LSD1-mediated crosstalk between phospholipid metabolism and histone methylation in mice with experimental myocardial infarction. J Mol Cell Cardiol.

[231] Choi SY, Kee HJ, Sun S, Seok YM, Ryu Y, Kim GR (2019). Histone deacetylase inhibitor LMK235 attenuates vascular constriction and aortic remodelling in hypertension. J Cell Mol Med.

[232] Han Y, Sun HJ, Tong Y, Chen YZ, Ye C, Qiu Y (2019). Curcumin attenuates migration of vascular smooth muscle cells via inhibiting NFκB-mediated NLRP3 expression in spontaneously hypertensive rats. J Nutr Biochem.

[233] Peng B, Peng C, Luo X, Wu S, Mao Q, Zhang H (2021). JNK signaling-dependent regulation of histone acetylation are involved in anacardic acid alleviates cardiomyocyte hypertrophy induced by phenylephrine. PLoS One.

[234] Ngo V, Fleischmann BK, Jung M, Hein L, Lother A (2022). Histone deacetylase 6 inhibitor JS28 prevents pathological gene expression in cardiac myocytes. J Am Heart Assoc.

[235] Okabe K, Matsushima S, Ikeda S, Ikeda M, Ishikita A, Tadokoro T (2020). DPP (Dipeptidyl Peptidase)-4 inhibitor attenuates ang II (Angiotensin II)-induced cardiac hypertrophy via GLP (Glucagon-Like Peptide)-1-dependent suppression of nox (nicotinamide adenine dinucleotide phosphate oxidase) 4-HDAC (histone deacetylase) 4 pathway. Hypertension.

[236] Ryu Y, Kee HJ, Sun S, Seok YM, Choi SY, Kim GR (2019). Class I histone deacetylase inhibitor MS-275 attenuates vasoconstriction and inflammation in angiotensin II-induced hypertension. PLoS One.

[237] Jung H, Lee E, Kim I, Song JH, Kim GJ (2019). Histone deacetylase inhibition has cardiac and vascular protective effects in rats with pressure overload cardiac hypertrophy. Physiol Res.

[238] Kim GJ, Jung H, Lee E, Chung SW (2021). Histone deacetylase inhibitor, mocetinostat, regulates cardiac remodelling and renin-angiotensin system activity in rats with transverse aortic constriction-induced pressure overload cardiac hypertrophy. Rev Cardiovasc Med.

[239] Wang K, Tang R, Wang S, Xiong Y, Wang W, Chen G (2022). Isoform-Selective HDAC Inhibitor Mocetinostat (MGCD0103) Alleviates Myocardial Ischemia/Reperfusion Injury Via Mitochondrial Protection Through the HDACs/CREB/PGC-1α Signaling Pathway. J Cardiovasc Pharmacol.

[240] Hu C, Peng K, Wu Q, Wang Y, Fan X, Zhang DM (2021). HDAC1 and 2 regulate endothelial VCAM-1 expression and atherogenesis by suppressing methylation of the GATA6 promoter. Theranostics.

[241] Xie W, Schultz MD, Lister R, Hou Z, Rajagopal N, Ray P (2013). Epigenomic analysis of multilineage differentiation of human embryonic stem cells. Cell.

[242] Chen T, Dent SY (2014). Chromatin modifiers and remodellers: regulators of cellular differentiation. Nat Rev Genet.

[243] Huang S, Shao T, Liu H, Wang Q, Li T, Zhao Q (2022). SIRT6 mediates MRTF-A deacetylation in vascular endothelial cells to antagonize oxLDL-induced ICAM-1 transcription. Cell Death Discov.

[244] Kusuyama J, Makarewicz NS, Albertson BG, Alves-Wagner AB, Conlin RH, Prince NB (2022). Maternal exercise-induced SOD3 reverses the deleterious effects of maternal high-fat diet on offspring metabolism through stabilization of H3K4me3 and protection against WDR82 carbonylation. Diabetes.

[245] Chapski DJ, Cabaj M, Morselli M, Mason RJ, Soehalim E, Ren S (2021). Early adaptive chromatin remodeling events precede pathologic phenotypes and are reinforced in the failing heart. J Mol Cell Cardiol.

